# Microglia and Stem-Cell Mediated Neuroprotection after Neonatal Hypoxia-Ischemia

**DOI:** 10.1007/s12015-021-10213-y

**Published:** 2021-08-11

**Authors:** Catherine Brégère, Bernd Schwendele, Boris Radanovic, Raphael Guzman

**Affiliations:** Department of Biomedicine and Department of Neurosurgery, Faculty of Medicine, University Hospital Basel, Basel, Switzerland

**Keywords:** Neonatal hypoxic-ischemic brain injury, Cerebral palsy, Neuroinflammation, Microglia, Stem cell therapy, Neuroprotection

## Abstract

Neonatal hypoxia-ischemia encephalopathy (HIE) refers to a brain injury in term infants that can lead to death or lifelong neurological deficits such as cerebral palsy (CP). The pathogenesis of this disease involves multiple cellular and molecular events, notably a neuroinflammatory response driven partly by microglia, the brain resident macrophages. Treatment options are currently very limited, but stem cell (SC) therapy holds promise, as beneficial outcomes are reported in animal studies and to a lesser degree in human trials. Among putative mechanisms of action, immunomodulation is considered a major contributor to SC associated benefits. The goal of this review is to examine whether microglia is a cellular target of SC-mediated immunomodulation and whether the recruitment of microglia is linked to brain repair. We will first provide an overview on microglial activation in the rodent model of neonatal HI, and highlight its sensitivity to developmental age. Two complementary questions are then addressed: (i) do immune-related treatments impact microglia and provide neuroprotection, (ii) does stem cell treatment modulates microglia? Finally, the immune-related findings in patients enrolled in SC based clinical trials are discussed. Our review points to an impact of SCs on the microglial phenotype, but heterogeneity in experimental designs and methodological limitations hamper our understanding of a potential contribution of microglia to SC associated benefits. Thorough analyses of the microglial phenotype are warranted to better address the relevance of the neuroimmune crosstalk in brain repair and improve or advance the development of SC protocols in humans.

**Graphical abstract:**

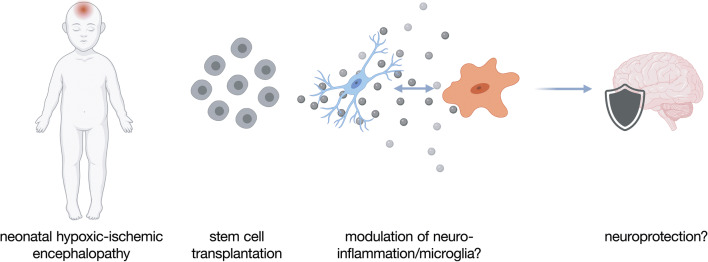

## Introduction

Perinatal hypoxic-ischemic (HI) brain injury is the result of a decreased blood and/or oxygen supply to the brain, namely asphyxia. It can occur in preterm and term infants (see Box 1 on definitions), in which latter case the condition is termed neonatal HI encephalopathy (HIE). The risk factors of hypoxia-ischemia are multiple, and include fetal, maternal, or placental conditions [1]. The incidence of HIE is estimated to be around 1.5 per 1000 live births in developed countries [2], and it is an important cause of mortality in neonates [3]. Surviving infants, depending on the severity of HI and maturational state of the brain, may suffer lifelong neurological sequelae, such as hearing and visual impairments, sensorimotor or learning disorders, seizures, and cerebral palsy (CP) [4].

At present, therapeutic hypothermia (TH) is the only approved intervention for term neonates diagnosed with moderate or severe HIE; it requires to be initiated within six hours after birth, and consists of either whole-body cooling or selective head cooling for up till 72 h to reach a body core temperature of 34 ± 0.5 °C. Randomized controlled trials of TH demonstrate a reduction in mortality without an increase in major disabilities in surviving infants at 18–22 months [5], but the narrow time window of application and incomplete efficacy of TH call for the development and evaluation of complementary and/or alternative interventions.

Stem cell (SC) treatment represents such a promising therapeutic option, as it allows for a wider therapeutic window in comparison to TH [6], and therefore is more clinically relevant. Preclinical studies indicate that SC based therapy can ameliorate or modify diverse aspects of HI-induced cerebral damages such as infarct size, apoptosis levels, axonal sprouting, neurite outgrowth, microglial activation, and provide some degree of functional improvements (e.g. motor, sensorimotor and cognitive behaviors) [7–10]. The results of SC based clinical trials for CP are also encouraging and show a modest yet significant effectiveness in improving gross motor function [11]. While the underlying biological mechanisms still remain elusive, the modulation of the immune system, in the central nervous system (CNS) and/or in the periphery is often put forward as a major mechanism responsible for this therapeutic benefit. Neonatal HI indeed elicits a sterile immune response that plays a crucial role in the progression of HI-induced injury. The innate immune system is activated from the very early phases until the tertiary phase of HI. In the brain, this process involves principally microglia—the resident myeloid cells—and astrocytes, which are also immune competent cells. Mast cells have also been shown to contribute to the neuroinflammatory response [12, 13]. A role for various peripheral innate immune cells into HI cerebral damage has also been demonstrated, in particular for monocytes that can infiltrate the brain parenchyma [14, 15], as well as for neutrophils [16–18] and natural killer cells [17, 19]. An adaptive immune response implicating T cells also seems activated after neonatal HI [17, 20, 21], but it is less characterized than that of the innate immune arm. Purinergic signaling (e.g. ATP/inflammasome axis [22]), specific cytokines, chemokines and their downstream signaling pathways also play a role in sterile inflammation and in the evolution of HI-induced cerebral injury [17, 23–32]. The complexity and multiplicity of all these inflammatory components has been the subject of recent extensive and comprehensive reviews [33–36]. Thus, to avoid redundancy, a particular emphasis is placed on discussing the microglial phenotype after neonatal HI in light of recent advances in the field, and the impact of developmental age at time of injury on the microglial response. The link between microglia and brain repair is then assessed by reviewing whether and how immune related treatments and stem cell therapy provide neuroprotection and impact microglia. Finally, the clinical evidence for immune modulation by SC and association with neuroprotection and neuroregeneration is discussed. The focus of this review being neuroinflammation/microglia and SC therapy in the context of HIE, only the findings from the corresponding postnatal days (P) 7–10 rodent model will be discussed.

Box 1 Definitions

**Table:** 

**Cerebral palsy (CP)** is a neurodevelopmental disorder. Its most recent definition is as follows [37]: “CP describes a group of permanent disorders of the development of movement and posture, causing activity limitation, that are attributed to nonprogressive disturbances that occurred in the developing fetal or infant brain. The motor disorders of cerebral palsy are often accompanied by disturbances of sensation, perception, cognition, communication, and behaviour, by epilepsy, and by secondary musculoskeletal problems.”	
**Prematurity** refers to any live birth before 37 weeks of gestation, and is further divided in moderate or late preterm (32 to <37 weeks), very preterm (28 to <32 weeks), and extremely preterm (<28 weeks) [38]. **Term pregnancy** for singleton until recently referred to 37-42 completed weeks of gestation. Due to heterogeneity in neonatal outcomes depending on gestational age within this five weeks’ time [39], the American College of Obstetricians and Gynecologists Committee has now refined the notion of term and suggests to use “full term“ for deliveries between 39 through 40 weeks of gestation [40]. This new definition of term pregnancy is not yet reflected in the clinical definition of neonatal HIE, which very often refers to term pregnancy as 36 weeks of gestation or more.	
**Human neonate** (synonym for newborn): the neonatal period corresponds to the first 28 days of life after birth.	
**Human infant**: corresponds to the period of time between birth and the first year.	
Where the Ambiguity Starts-Definition of Rodent Age	
**Rodent neonate**: there is a lack of consensus on the timeframe corresponding to the neonatal period. Depending on authors, the age range for a neonate is between birth and P10, or sometimes between birth and weaning, i.e. 21 days.	
**Rodent juvenile** (synonym for adolescent): juvenility refers to the transition between “childhood” and “adulthood”, during which many neurodevelopmental alterations are still occurring (maturation of cortical and limbic structures, of neurotransmitter systems); it associates with typical adolescent-like behaviors (i.e. risk-taking behavior, increased emotional reactivity). The time frame for juvenility is not strict; tentatively in rodents the time period between P28 and P42 has been suggested, but it can start before, and end after [41]. In contrast to juvenility, rodent puberty refers to the well-defined time period during which sexual maturity is achieved. It occurs at around P32-40 and P38-45 for rat females and males, respectively.	
**Rodents** may be considered as **adults** if they are at least 12 weeks of age [42].	

## Pathophysiology of Neonatal HI: A Complex and Evolving Brain Injury

Among neural cells, neurons are the most vulnerable to HI, due to their high energetic requirements to ensure neurotransmission and maintain ionic gradients across their membrane. Specific groups of neurons and regions (cortex, thalamus and putamen) in the developing brain have been shown to be particularly sensitive, a phenomenon referred to as selective vulnerability [43, 44]. Neuronal injury occurring after HI progresses over time, and based on investigations in animal models of neonatal HI and clinical observations, the following chronological phases have been proposed. The first phase is referred to as a primary energy failure (or acute phase), and immediately follows the HI insult. The drop in glucose and oxygen levels induced by HI causes a rapid reduction in ATP levels, which then leads to a failure of ATP dependent processes in neurons, in particular the transmembrane ion pumps. This energy failure triggers a cascade of toxic events, e.g. intracellular accumulation of sodium, calcium and water, brain acidosis, and eventually results in cytotoxic edema, extracellular accumulation of excitatory amino acids, and death of neurons, mainly through necrosis but also through apoptosis. Depending on the severity and duration of the HI episode, a latent phase may follow during which partial recovery of neuronal metabolism can occur; although its duration and exact timing is unknown, it is often considered that this phase is an appropriate time window to begin potential neuroprotective treatments, such as TH or SCs injection, in order to limit the ongoing toxic processes and prevent the progression towards the secondary phase. In the absence of intervention or if the primary phase is prolonged and severe, then the latent phase is short and a secondary delayed energy failure phase (six to 72 h after the insult) ensues, during which further deterioration and cell death will occur, characterized by excitotoxicity, mitochondrial failure, acute inflammation, oxidative stress, and increased seizure activity. Finally, there is evidence that some injury processes may persist over months or perhaps years, resulting in so called tertiary damages [45]; the suggested mechanisms underlying such damages include astrogliosis, persistent inflammation and epigenetic changes, all of which may lead to exacerbated cell loss and sensitization to potential second hits.

## Modeling HIE in Rodents: The Rice-Vannucci Model

The most widely used experimental model to study HIE is the Rice-Vannucci model [46]. Initially developed in the immature Sprague-Dawley rat, it was later adapted to the mouse [47, 48], thus allowing to study the contribution of individual or combination of genes to the HI-induced pathological processes using genetically modified mice. This model combines unilateral ligation of the common carotid artery (CCA) and subsequent exposure to 8–10% oxygen/balance nitrogen (hypoxia) for a duration of between 40 min up to 3.5 h. The time interval between permanent CCA ligation and subsequent exposure to hypoxia influences the development of brain injury in the rodent neonate. If initiated three to four hours after CCA ligation, hypoxia induces a discernible neurologic lesion histologically and a drop in ATP levels in the ipsilateral hemisphere. Nevertheless, the brain injury is almost absent if hypoxia is initiated 24 h post-ligation [49, 50]. This is most likely because collateral compensatory blood flow occurs within 24 h, as demonstrated in the adult rat in which one to two days and up to six weeks after CCA ligation, a significant enlargement of the anastomosis of the ipsilateral posterior communicating artery was measured [51, 52]. Hypoxia alone also does not cause obvious neuronal death, but rodent neonates exposed to global hypoxia between P7-P10 show a heightened susceptibility to provoked seizures, and develop spontaneous seizures shortly after hypoxia exposure, and during adulthood [53, 54]. Thus hypoxia-only models are valuable to study short and long-term consequences of neonatal epilepsy (reviewed in [54]).

The brain injury induced by HI is observed in the ipsilateral hemisphere, i.e. on the same side of carotid ligation, and generally leaves the contralateral hemisphere intact. Duration of hypoxia obviously influences the severity of lesion [55], but even when using the same exposure time to hypoxia within an experiment, animals still display variable brain damage, ranging from none, mild, moderate to almost fully infarcted ipsilateral hemisphere [56, 57]. The lesions are commonly observed in both grey and white matter, and affected structures include the cortex, striatum, thalamus and hippocampus. This neuropathology is accompanied by short-term sensorimotor deficits [58] and long-lasting motor, learning and memory impairments [59–61]. Of note, the HI-induced sensorimotor and motor deficits are lateralized, and observed on the contralateral limbs [62].

The HI surgery in rodents is typically performed at P7 in rats, and between P7–10 days in mice. While not explicitly stated in the original publications, the choice of these days was based on the work by Dobbing and Sands [63], who reported that the peak of the brain growth spurt occurred at P7 in the rat brain and around birth in humans (see Box 2 on the brain growth spurt). There is no definitive answer as to whether the degree of maturation of the rat brain at P7 day corresponds to that of the human brain at birth, because it mainly depends on the developmental indices considered. For instance, in terms of functional cortical development (measured by the number of synapses, electrical activity and activity of some neurotransmission system), the brain of a human full-term neonate resembles that of P12–13 rat [64]. When white matter maturation is considered, the human brain between 30 and 36 weeks of gestation then roughly corresponds to that of a P7 rodent brain [65]. Based on these criteria and others, some authors posit that the P7 rat brain may still be premature in comparison to that of a human term infant, and argue that the P10–11 stage may be more appropriate to study HIE and potential therapies [56, 66]. Notwithstandingly, the overwhelming majority of investigators—including ourselves—has and continues to use the classical P7 rat model and thus the prevalence of this model facilitates inter-studies comparisons (see all Tables). As models translating the timing of neurodevelopment events from humans to rodents are now available (http://translatingtime.org) [67–69], an informed choice can be made concerning the postnatal age at which HI is induced, keeping in mind that a three to four day shift may impact neuropathological and behavioral outcomes.

HI is also performed in rodent neonates younger than P7. As P1-P5, in terms of brain maturation, roughly corresponds to a human fetus between 24 to 32 weeks of gestation, HI induction at this postnatal age attempts to model brain lesions observed in the very preterm infant, in particular white matter damage and its most severe form periventricular leukomalacia (PVL). There is currently no single model of “encephalopathy of prematurity “[70], as the day of HI induction, the severity of hypoxia (typically 5% O_2_ because 8% O_2_ fails to induce neuronal damage [71]) and its duration, vary greatly between studies [72–77]. In addition, HI is sometimes combined with an inflammation trigger (e.g. LPS) [78, 79], since inflammation is thought to be a major cause of brain injury in preterm infants [80, 81]. The pattern of brain lesion overlaps to a certain extent with that of the P7 model, for instance alteration in cortical development and myelination can be observed, albeit with subtle differences in neuronal populations affected, and short as well as long-term sensorimotor, motor and cognitive defects are also observed [82–85]. Nevertheless, contrary to the HI-P7 model, the hippocampus is typically spared in neonates exposed to HI before P7 [71].

Box 2 Age Matters/the Brain Growth Spurt

**Table:** 

Age is admittedly a critical factor in the field of neurosciences [86], but even more so in neurodevelopment. Why do we model a brain injury occurring at around birth in humans in seven-day-old rodents? The answer is that the relation of birth to the degree of maturation of the brain differs between species. While humans, just as rodents, are considered an altricial species, their brain maturation at birth is actually more advanced than that of rodents.	
The Brain Growth Spurt	
The growth of the brain, in terms of weight, is not uniform across development. If age is plotted against brain weight (expressed as percent of adult value), it yields a sigmoid curve, whichever species considered. When derivated into a velocity curve, then a peak becomes visible: it corresponds to a phase of extremely fast growth, referred to as the brain growth spurt. A major difference between species is the timing of this growth spurt in relation to birth. In humans, there is a major brain growth spurt that begins at around mid-gestation, peaks at birth, and ends at around 3 to 4 years [87]; between birth and the age of 3-4 years, the weight of the human brain quadruples, reaching almost 80% of the adult brain weight. In the rat, the brain growth spurt is postnatal only: it starts at birth, peaks at postnatal day 7, and ends at around postnatal day 25. The timing of the peaks, birth in humans, and P7 in rats, is the basis for the P7 rat model of neonatal HI. It is still frequent to read in the literature that the developmental stage of the rat brain at P7 roughly corresponds to that of humans at term birth. Such a statement is yet a shortcut, as other developmental aspects beyond the growth spurt can also be considered, as discussed in the main text. Thus, the decision-making concerning the time point at which an injury is induced in rodent neonates to mimic a human condition will depend on the research question and should be based on the available neurodevelopmental data.	

## The Impact of Neonatal HI on Microglia/Macrophages

### Microglial Phenotype after Neonatal HI

In the healthy developing brain, microglia are implicated in various ongoing neural processes, such as synaptic pruning [88, 89], maintenance of normal brain structure and olfaction [90], phagocytosis of dead cells and debris, but also of viable neural precursor cells in the subventricular zone (SVZ) [91], axon guidance [92], as well as neurogenesis and oligodendrogenesis [93] .

Experimental data in rodents indicate that neonatal HI induces early (two hours post-HI) and persisting (up until 17 days) phenotypical changes in microglia in the ipsilateral hemisphere, including an increase in cell count, a spectrum of morphological changes from a ramified to an amoeboid state, and an elevated expression of markers of microglia activation (OX-6, major histocompatibility complex II; OX-18, major histocompatibility complex I; CD68) [94, 95] (see Table 1 for an overview on microglia and other cerebral immune-related findings in the rodent model of neonatal HI). The time course of these changes is region-dependent, with the hippocampal microglia showing earlier activation than their cortical, striatal and white matter counterparts [32, 96]. There is a commonly held view that in the earlier phases of HI injury, microglia adopt a M1 “classically activated” pro-inflammatory phenotype, while later on switch to a M2 “alternatively activated” anti-inflammatory phenotype (see Boxes 3 and 4 for a historical perspective and a summary on the M1/M2 concept). Thus, a study reported that M1 microglia predominate in the ipsilateral hemisphere three hours after neonatal HI, while at 24 h, both M1 and M2 microglia can be detected, suggesting a switch in the inflammation state of the brain [31]. Nevertheless, a limitation in this study is that a single marker for each state was used (iNOS for M1, and CD206 for M2). A more recent study revealed a slightly more complex picture, with higher and concomitant expression of both anti-inflammatory and pro-inflammatory genes in the ipsilateral than in the contralateral hemisphere [97]. In addition, FACS analysis of the CD11b^+^ population (microglia/macrophages) for CD86 (classical) and CD206 (alternative) cell markers revealed an expanded and predominant population of CD11b^+^CD86^+^CD206^−^ cells in the injured hemisphere over several days post injury, a relative suppression of CD11b^+^CD86^+^/-CD206^+^ cells, and a small “non-polarized” cell population expressing neither CD86 and CD206, but expressing galectin-3, an immunomodulatory mediator. Our own investigations of the microglial phenotype in the SVZ—three and 13 days after neonatal HI in rat—also converge towards the same point since CD11b + microglia isolated from the ipsilateral subventricular zone (SVZ) of HI-exposed rat neonates upregulate both pro- and anti-inflammatory genes until 13 days post-injury, and do not differentially express markers of acute inflammation such as IL-1β, IL-6, TNF-α, and IFN-γ when compared to microglia isolated from the SVZ from sham animals [98]. This underlies the specificity of the SVZ microglial response versus that observed in regions nearby the ischemic core. Further comparative analyses of our microglial gene datasets with published ones revealed that, three days after neonatal HI, the transcriptome of the SVZ microglia from HI-exposed animals does not fit a M1 or M2 state, but rather present similarities with that of primed microglia isolated from mouse models for aging and neurodegenerative diseases, characterized by an altered immune profile associated with phagocytic clearance and possibly neurotrophic features [99]. Thus the concept of “disease-associated microglia” may apply beyond the neurodegenerative environment [100, 101]. Altogether, these data uncover a previously unrecognized microglial diversity/complexity after neonatal HI that surpasses the traditional M1/M2 frame of thought [102, 103].

**Table 1:** Cerebral immune-related findings in the rodent model of neonatal HI

Reference	Species and Model of HI	Immune-related findings in the ipsilateral (Il) brain in comparison to the contralateral (Cl) brain	Conclusion(s)
McRae A et al., 1995 PMID: 7743644	WFU rat P7 Left CCA ligation 100 min 7.7% O_2_ Sacrifice time-points: 2–3 h, 24 h, 2–4 d and 2 w	*Immunohistochemistry using OX-18 (Ab against rat MHC-Class I antigens), OX-42 (anti-CD11b/c, a component of complement receptor 3 or CR3) and OX-6 (Ab against rat MHC-Class II antigens)* 2–3 h: - Corpus callosum: slight increase in OX-18 immunoreactivity. - Thalamus: scant OX-18/OX-42 immunoreactivity. - Cortex or striatum: absence of OX-18^+^ microglia. - No OX-6 immunoreactivity in any region. 1 d: - Corpus callosum: OX-18 and OX-42 immunoreactivity increases. - No OX-6 immunoreactivity in any region. 2–4 d: - Cerebral cortex, striatum and corpus callosum: peak in OX-18 and OX-42 stainings. - Thalamus: scant OX-18/OX-42 immunoreactivity. - OX-6 immunoreactivity detected. 2 w: - OX-18/OX-42 immunoreactivity persists at a high level in the cortex and striatum; the immunoreactivity is lost in the corpus callosum. - Thalamus: peak in OX-18/OX-42 immunoreactivity. - No OX-6 immunoreactivity in any region.	- The loss of MAP2 precedes microglial activation in cortex and striatum. - The time course of microglial activation depends on the brain region considered. - Overall, in comparison to the Cl hemisphere, a robust proliferation of microglia starts at 24 h, peaks at 2–4 days, and persists up to 14 d post-HI.
Ohno M et al., 1995 PMID: 7743650	Sprague-Dawley rat P7 Left CCA ligation 120 min 8% O_2_ Sacrifice time-points: 3, 6, 12, 24, 48 and 72 h and 5 and 14 d	*Immunohistochemistry using OX-42, ED-1 (anti-CD68 Ab) and ED-2 (anti-CD163 Ab, marker of the monocyte/macrophage lineage)* - Cerebral cortex: intense OX-42 immunoreactivity evident 24 h after HI. - Maximal microglia/macrophage reaction 5 d after HI. - Positive OX-42 staining still detected in cortex and hippocampus 14 days after HI. - CD68^+^ cells become visible after 48 h. - No change in ED-2 immunoreactivity observed in the HI brain.	Microglial response to HI appears to be the most prominent among glial cells.
Szaflarski J et al., 1995 PMID:7762028	Sprague-Dawley rat P7 Right CCA ligation 180 min 8% O_2_ Sacrifice time-points: 1, 2, 4, 8, 12, 24, and 48 h	*RT-PCR* - In the Il cortex, hippocampus and striatum, a peak in IL-1β mRNA expression occurs 4 h post-HI; return to normal levels by 24 h. - Similar transient increase in TNF-α mRNA.	The elevation in the gene expression of pro-inflammatory cytokines suggests a potential functional role in perinatal brain injuries.
Hagberg H et al., 1996 PMID: 8888290	Wistar rat P7 Left CCA ligation 70–100 min 7.7% O_2_ Sacrifice time-points: 1, 3, 6, 10, 20, and 48 h, and at 14 d	*Cytokines/gene expression* No IL-1 bioactivity detectable in the brain from control animals. After HI: - In the Il hemisphere, IL-1 bioactivity is undetectable 1 h after the insult, increases at 3 h, peaks at 6 h, and remains significantly higher than in Cl hemisphere up to 48 h. - IL-6 bioactivity and mRNA are also increased in response to HI with a similar time course to IL-1, but the changes are not as pronounced.	IL-1 plays a role in the pathophysiology of neonatal HI.
Ivacko JA et al., 1996 PMID: 8825384	Sprague-Dawley rat P7 Right CCA ligation 180 min 8% O_2_ Sacrifice time-points: 10 min to 5 d	*G. simplicifolia B4-isolectin histochemistry* - In the hippocampus, evidence of microglia activation based on morphological changes 10 min after HI. - In the hippocampus, thalamus, cortex and striatum, increased number of activated microglia 4 h after HI. - Even more extensive microglial activation 8 h post-HI followed by progressive increase at 24, 48 and 72 h.*ED-1 (CD68) immunohistochemistry* - No increase 10 min post-HI, but immunoreactivity detectable 4 h post-HI in the Il ependyma and choroid plexus. At 24 h post-HI, reactive cells infiltrate the pyramidal cell layer and at 48 h, there is diffuse infiltration throughout the hippocampus.	- Based on lectin and ED-1 labelings, the density of microglia activation peaks between 2 and 4 d after HI. - 5 d post-HI, microglial activation is less intense in cortex, striatum and hippocampus. - The regional distribution of microglia activation generally corresponds to that of the histopathology. - Overall, microglia activation occurs very early in the forebrain after HI; it peaks around 2 d post-HI, and begins to wane 4 d post-HI.
Hudome S et al., 1997 PMID: 9128280	Wistar rat P7 Right CCA ligation 135 min 8% O_2_ Sacrifice time-points: 0, 5, and 30 min, and 2, 4, 8, 18, and 42 h	*Hemispheric myeloperoxidase (MPO) activity* MPO activity in both hemispheres of HI-exposed animals is higher than in control animals at all times of sacrifice, with a peak between 4 and 8 h post-HI. There is no difference in MPO activity between Il and Cl from HI-injured rats. *Neutrophil staining with ANS (anti-neutrophil serum) in the brain* The count of neutrophils in HI-Il and HI-Cl hemispheres does not differ significantly from that in normal brain tissue. Overall there are significantly more neutrophils in the Il than in the Cl hemisphere. Staining indicates that the majority of neutrophils are located within the leptomeningeal blood vessels on the surface of the brain or within the choroid plexus, thereby indicating that the neutrophils do not extravasate from blood to the brain parenchyma.	Neutrophils may play a role in the pathogenesis of neonatal HI, via yet unclear mechanisms.
Ivacko JA et al., 1997 PMID: 9270493	Sprague-Dawley rat P7 Right CCA ligation 180 min 8% O_2_ Sacrifice time-points: • For in situ hybridization: 0, 1, 2, 4, 8, 16, 24, 48, and 120 h • For immunocytochemistry: 10 min, 2.5, 4, 8, 12, 24, 48 and 120 h	In situ *hybridization and immunohistochemistry for MCP-1 (monocyte* *chemoattractant* *protein-1, or CCL2, a potent chemotactic factor for monocytes; the receptor is CCR2)* MCP-1 mRNA and protein peak within the first 24 h in the lesioned hemisphere and are no longer detected after 48 h. *G. simplicifolia B4-isolectin histochemistry* In the lesioned forebrain structures, the microglial/monocyte response (i.e. accumulation and morphological transition to an activated state characterized by cell body enlargement and shortened, thickened cell processes) peaks at about 48 h after HI, plateaus from 48 to 96 h, and then begins to wane. Many activated microglia and/or monocytes are still detectable throughout the Il cerebral hemisphere 5 to 6 d after lesioning.	- HI induces MCP-1 gene and protein expression in the Il hemisphere within 48 h, after which time point MCP-1 protein declines. - MCP-1 immunoreactive cells appear to be neurons. - Microglia express MCP-1 receptors. The microglial response is more prolonged than that of MCP-1 after HI. -- Overall, MCP-1 may play a role in the microglial response; however it should be noted that microglial response is observed prior to detection of MCP-1 mRNA or protein.
Bona E et al., 1999 PMID: 10203141	Wistar rats P7 Left CCA ligation 70 min 7.7% O_2_ Sacrifice time-points: 3 h, 6 h, 12 h, 24 h, 72 h, 7 d, 14 d and 35 d	*RT-PCR* Whole Il hemisphere in comparison to Cl: early induction of α (gro and MIP-2) and β (MIP-1α and MIP-1β) chemokines (within 6 h); induction of TNFα (peak at 12 h) and Il-1β (peak at 6 h). *Hematoxylin and eosin staining* Increased neutrophil count at 12 h post-HI, then return to baseline levels; neutrophils found within and around vessels in the damaged gray and white matter. *Immunohistochemistry* - 3 d after HI, increase in CD4 expression bilaterally in corpus callosum, and in damaged regions with a peak 7 d post-HI; Persisting CD4 expression in injured regions up to 14–35 d post-HI. - At 24 and 72 h post-HI, discrete increase in CD8+ cells in the CC and in the HI damaged regions. - At 12 h post-HI, variable increase in NK+ cells immunoreactivity in the Il hemisphere, with the number of NK cells ranging from 15 to 20 to 60. This accumulation of NK cells is observed in the infarcted area, and appears to persist up till 14 d post-HI. - OX-18+ cells (microglia/macrophages) detectable as early as 3 h post-HI, and increase up till 72 h post HI, with the most pronounced induction in the severely HI-affected brains. - Marked increase in β2-integrin immunoreactivity in the infarct region 7 d post-HI that persists up to 35 d.	- There is an early induction of chemokine gene expression after HI that precedes immune cells activation. - The activation of microglia/macrophages (MHC-Class I staining), CD4 lymphocytes and neutrophil accumulation persist for up to 35 d post-HI. - Overall, chemokines potentially play a role in initiating the inflammatory response associated with HI.
Liu X et al., 1999 PMID: 10532634	WT and ICE-deficient mice on a mixed 129/Sv and C57BL/6 background P9 -P10 Right CCA ligation 70 min 10% O_2_: moderate HI 120 min 10% O_2_: severe HI Sacrifice time-points: 5 or 21 d	- Attenuation of HI-induced brain damage in ICE deficient mice (ICE = Interleukin-1 converting enzyme; animals are deficient in systemic IL-1α and IL-1β) in comparison to WT mice if animals undergo moderate HI - However, the degree of infarct severity in both ICE and WT mice remains the same if animals are subjected to severe HI.	- Neonatal ICE-deficient mice are resistant to a moderate HI insult, but not to a severe insult. - Overall, the activity of ICE contributes to the progression of neonatal HI injury to some extent.
Xu H et al., 2001 PMID: 11376856	WT and ICE-deficient mice on a mixed 129/Sv and C57BL/6 background P9 -P10 Right CCA ligation 10% O_2_, 40 min (minimal) or 70 min (moderate) or 120 min (severe) HI insult Sacrifice time-points: 0, 8, 16, 24 and 48 h	*mRNA and protein levels of MCP-1, and mRNA levels of its receptor CCR2* - HI markedly stimulates MCP-1 expression both in ICE deficient mice and in wildtype mice at 8 h post-HI. - In animals subjected to the mildest HI protocol, the Il hemisphere concentration of MCP-1 is substantially lower in the ICE deficient mice than in the wildtype group. - The severity of the HI insult influences the magnitude of the MCP-1 response. - CCR2 mRNA is detected only at 24 h post-HI, and expression is equivalent between HI-exposed ICE deficient mice and HI-exposed wildtype mice.	- HI stimulates MCP-1 expression in the brain even in the absence of IL-1β activity. - The activity of ICE participates to the regulation of the acute inflammatory response to HI brain injury in neonatal mice.
Cowell RM et al., 2002 PMID: 11872906	Sprague-Dawley rat P7 Right CCA ligation 60 to 150 min 8% O_2_ Sacrifice time-points: 0, 4, 8, 16, 72 and 120 h	*Immunohistochemistry for MIP-1α,* *macrophage* *inflammatory* *protein, a monocyte chemoattractant protein that belongs to the β-chemokine family* - In normal P7 to P12 rat brain, MIP-1α is found in physiologically activated microglia. - From 8 to 120 h post-HI, MIP-1α immunoreactive cells are detected in the injured cortex, hippocampal dentate gyrus and thalamus, but not in the corresponding Cl regions; they are associated with capillaries. - No increase in immunoreactivity for the 2 receptors of MIP-1α, CCR1 and CCR5, is detectable. *ELISA* MIP-1α concentration peaks in the Il hemisphere at 16 h post-HI and declines thereafter.	- Neonatal HI induces expression of MIP-1α in the monocyctic/macrophage lineage. - Overall, MIP-1α contributes to neonatal HI-induced inflammation.
Hedtjärn M et al., 2002 PMID: 12122053	Wistar rat P7 Left CCA ligation 60 min 7.7% O_2_ Sacrifice time-points: 1, 3, 6 and 14 d	*Immunohistochemistry* - Immunoreactivity for IL-18 (member of the IL-1 family) is observed in injured areas in cortex and thalamus in the Il hemisphere at 6 and 14 d after HI. - Cells expressing IL-18 are identified as microglia and they co-localize with caspase-1 positive cells. - IL-18R immunoreactivity is primarily found on neurons: the expression remains similar between control and HI-exposed animals, and no difference can be observed between Il and Cl hemispheres. *RT-PCR* Between 3 h and 14 d post-HI, continuous and significant increase in mRNA expression of IL-18 and caspase-1 in Il versus Cl hemisphere of HI-exposed animals. *Cytokines (ELISA)* - Increase in active IL-18 protein levels in the injured hemisphere 3 and 6 d after injury. - In contrast, the maximal increase in IL-1β protein occurs 8 h post-HI, earlier than the peak in caspase-1 and IL-18 protein expression, and then a secondary rise occurs at 6–14 d after HI.	The relative resistance of IL-18 deficient mice to HI injury suggests the involvement of IL-18 in the pathogenesis of neonatal HI.
	C57BL/6 wild-type mice and IL-18 KO mice P9 Left CCA ligation 60 min 7.7% O_2_ Sacrifice time-point: 3 d	*Immunohistochemistry* - The area of infarction measured as lack of MAP2 immunoreactivity is significantly reduced in IL-18 KO compared to wildtype mice. - The neuropathological scoring of HI brain injury is also lower in IL-18 KO mice.	
Hedtjärn M et al., 2004 PMID:15625408	C57BL/6 wild-type mice P9 Left CCA ligation 60 min 10% O_2_ Sacrifice time-points: 2, 8, 24, and 72 h	*Microarray global inflammatory gene expression analysis in the developing brain* - 148 inflammatory genes are differentially expressed 2 to 72 h after HI; 97% of the genes are up-regulated. - The differentially regulated genes are divided in 9 functional categories based on function and cellular expression: chemokines, complement-related genes, genes expressed by leukocytes, macrophage related genes, genes related to B and T-lymphocytes, genes induced or regulated by interferons, MHC class I- and II related genes, others genes involved in immune and inflammatory responses and genes related to adhesion.	- The immune-related response in the brain after neonatal HI is complex and involves many immune cells, microglia macrophages, T and B-cells, NK cells, mast cells dendritic cells and leukocytes. - Both pro-inflammatory and anti-inflammatory cytokines are induced.
Hedtjärn M et al., 2005 PMID:16046848	C57BL/6 mice IL-1β 18 double-knockout mice and mice lacking the gene for IL-1β while being heterozygous for IL-18 IL-1αβ double knockout mice P9 Left CCA ligation 60 min 10% O_2_ Sacrifice time-point: 1 w	- When overall brain injury is measured, mice deficient in IL-1β in combination with IL-18 are moderately protected. - Mice deficient in either IL-1β, or IL-1β in combination with IL-1α, are not protected against neonatal HI.	IL-18, but not IL-1α and IL-1β, contributes to neonatal HI-induced neuronal injury.
Van Den Tweel ER et al., 2006 PMID: 16492985	Wistar rat P12 Right CCA ligation 90 min 8% O_2_ Sacrifice time-points: 30 min to 48 h	*Cytokines* - The mRNA of TNF-α, TNF-β, and IL-1β are detected 6 h post-HI and peak 24 h after HI. - After 48 h, mRNA expression for the cytokines return to the levels measured at 6 h. - A large individual variation in the cytokine levels after HI is noted. - No significant differences are observed between expression of the cytokines in the Il and Cl hemispheres. - Hypoxia alone induces an increase in IL-1β mRNA expression in both hemispheres. *Immunohistochemistry for CD68* - The number of CD68+ cells in the Il hemisphere starts to increase at 12 h and continues to rise until 48 h post-HI. - No change in the number of CD68+ cells in the CL hemisphere is observed.	- There are bilateral increases in mRNA of cytokines after HI, thus indicating that hypoxia alone is sufficient to induce these changes. - It is suggested that changes that occur bilaterally are not toxic per se, but require the ischemic milieu to exert neurotoxic effects.
Jin Y et al., 2009 PMID: 19520991	Wistar rat P7 Right CCA ligation 75 min 8% O_2_ Sacrifice time-points: 0, 1, 2, 4, 24 and 48 h; 1, 2 and 4 w	*Toluidine blue histology to identify mast cells (MCs)* - Rapid increase in MCs in the Il hemisphere in comparison to both control and Cl hemispheres immediately after HI and throughout the initial 48 h period. *Impact of cromolyn treatment, a MC stabilizer*	Mast cells appear to contribute to theinflammatory response to neonatal HI andcould be the initiators of the immuneresponse.
Winerdal M et al., 2012 PMID: 22567156	C57/bl6 mice P10 Right CCA ligation 60 min 10% O_2_ Sacrifice time-points: 1, 2, 7, 14, 90 and 190 d	*FACS* - In comparison to sham and Cl hemisphere, the number of CD11b^+^/CD86^+^ increases 1 d post HI, then declines, peaks again 7 d post-HI and then returns to control levels 2 w post-HI. - MHC-II expression in CD11b^+^ cells is increased 1 d post-HI, then declines until peaking again 3 mo post-HI and returns thereafter to baseline levels. - Two peaks of CD4^+^ T-lymphocytes influx in the HI hemisphere are observed, one at 1 w post-HI and the second one at 2 w post-HI. - An influx of CD8^+^ cytotoxic T-cells is observed 2 w post-HI, elevated numbers are still present at 3 mo and then return to baseline levels at 7 mo post-HI. - The T cells entering the injured brain hemisphere 1 w after HI express the naïve T cell marker CD45rb, but only few CD45rb^+^ cells are observed 3 mo post-HI. - The expression of CD69 and CD25 (activation markers of lymphocytes) increases both in CD4^+^ and CD8^+^ T-lymphocytes to reach maximum levels 3 mo post-HI. - A subpopulation of CD4^+^CD8^+^ double positive T-cells, not seen in shams, is present in the HI hemisphere at 3 mo but disappears at 7 mo.	- After neonatal HI, the first immune activation is that of CD11b^+^ cells in the brain and correspond to microglia. - The activation of microglia may prepare for the activation of adaptive immune response. - It is hypothesized that the infiltrating T-cells 1 w after HI remain in the brain, and then become activated. - The persistent immune activation of T-lymphocytes months post-HI may elevate the risk of autoimmune brain damage later in life.
Bonestroo H et al., 2013 PMID: 23689428	Wistar rat P7 Right CCA ligation 90 min 8% O_2_ Sacrifice time-points: 3 or 24 h	*Immunohistochemistry in the brain* - iNOS+-Iba1+ cells: 3 h post-HI, small number in cortical regions and 24 h post-HI, significant increase in the number of these cells throughout the Il cortex, striatum, thalamus and small increase in the hippocampus. - CD206 + -Iba1+ cells: no significant increase 3 h post-HI, but significant increase 24 h post-HI in the cortex, thalamus and hippocampus. *MPO activity assay in the brain as a measure of neutrophil influx* - 3 h post-HI, slight and nonsignificant increase in neutrophils within meninges and cortex. - 24 h post-HI, significant increase in neutrophils throughout cortical and hippocampal parenchyma. *Gene expression* 3 h post-HI in the brain: Upregulation of the pro-inflammatory cytokines TNF-α and IL-1β in the Il hemisphere in comparison to levels in sham animals; the anti-inflammatory cytokine IL-10 is significantly upregulated; also increase in CINC-1 and MCP-1; no change in TGF-β, IL-4 or IL-6. 3 h post-HI in the liver: Downregulation of TNF-α and IL-1β compared to levels in sham animals. Trend towards IL-10 upregulation; increase in CINC-1, but decrease in MCP-1; no change in TGF-β, IL-4 or IL-6. 24 h after insult in the brain: TNF-α, MCP-1 and CINC-1 still elevated in the Il hemisphere compared to levels in sham animals; IL-1β and IL-10 normalized 24 h after HI; no significant changes in IL-4 or IL-6. 24 h after insult in the liver: Significant decrease in IL-10 and MCP-1, but no other difference between HI and sham animals.	- Neonatal HI induces early pro-inflammatory response in the brain with a concomitant increase in influx of neutrophils; microglia show a “M1-like phenotype “3 and 24 h post-HI. - The inflammatory state of the brain may change 24 h post-HI: the anti-inflammatory cytokine TGF-β increases and macrophages/microglia may adopt the “M2-like phenotype“. - The nature of the inflammation induced by HI in the liver differs from that observed in the brain: a downregulation of pro-inflammation mediators is observed 3 h post HI; 24 h post-HI, the hepatic cytokine/chemokine expression levels are back to these measured in control animals. - Overall, the authors suggest that the cerebral inflammation induced by HI is responsible for the downregulation of the hepatic pro-inflammatory cytokine response.
Furukawa S et al., 2014 PMID: 24680905	Wistar rat P7 Left CCA ligation 120 min 8% O_2_ Sacrifice time-points: 24, 48 and 72 h	*Immunohistochemistry using tomato (Lycopersicon esculentum) lectin* - In the hippocampus, activation of microglia is strong for the first 48 h after HI and then decreases. - In the cortex and white matter, activation of microglia is weak at 24 h and then becomes strong over 48 and 72 h. *ELISA for TNF-α in Il hemisphere* At all time points examined after HI, TNF-α concentration remains low and unchanged.	The temporality of microglia activation after neonatal HI is region-specific: hippocampal microglia appear to respond first, followed by white matter and cortical microglia.
Hellström Erkenstam N et al., 2016 PMID: 28018179	C57BL/6 mice P9 Left CCA ligation 50 min 10% O_2_ Sacrifice time-points: 6 h, 1, 3 and 7 d	*qRT-PCR of total cortical homogenates* - 6 h after HI, concurrent upregulation of genes related to classical (CD86, IL-6, IL-1β, Cox2, iNOS) and alternative (CD206, IL-10, Fizz1, Arg1) activation. No significant upregulation in these genes at later time points. *FACS analysis of the CD11b*^*+*^ *populations* - In the Il hemisphere, the total population of CD11b^+^ cells increases at 24 h, 72 h and 7 d after HI, with the largest increase occurring at 24 h. - Similarly, the different CD11b^+^ cell populations expand at the same time points, with the largest expansion also observed at 24 h. - The pool of classically polarized-like cells (CD11b^+^CD86^+^CD206^−^) is the dominant cell type at 24 h post-HI. - The proportion of alternatively polarized-like cells (CD11b^+^CD86^±^ CD206^+^) cells never exceeds 8% of the total CD11b^+^ population, in either the Cl or the Il hemisphere, at any time point examined. - In the Cl hemisphere, the unpolarized CD11b^+^CD86^−^CD206^−^ population is the largest population (approximately 80% of all CD11b^+^ cells). *Immunoassay for Galectin-3 protein expression in lysates of FACS sorted cell populations* - The 3 different CD11b^+^ cell populations show increased galectin-3 expression at different time points post-HI: at 1 and 3 d for the CD86^−^CD206^−^ population, at 7 d for the CD86^+^CD206^−^ population, and at 3 d for the CD86^±^ CD206^+^ population. - CD11b^+^CD86^−^CD206^−^ cells are the main source of increased galectin-3 protein in the injured hemisphere at all three time points.	- The data illustrate that the traditional concept of “classical” versus “alternative” activation of microglia/macrophages cannot be applied to the complex phenotype observed in vivo after neonatal HI. - It is the unpolarized population of CD11b^+^ cells that express the most galectin-3, an immunomodulatory factor. These cells may play a role in the recruitment of cells in the brain and in the immune response after neonatal HI.
Smith P et al., 2018 PMID: 30376851	Lys-EGFP-ki mouse: marking of peripheral mature myeloid cells (monocytes, monocyte-derived macrophages and granulocytes) P9 Left CCA ligation 50 min 10% O_2_ Sacrifice time-points: 6 h, 1, 3, 7, 14 and 28 d	*Flow cytometry* - CD11b + EGFP+ infiltrating myeloid cells are significantly increased in the ipsilateral compared with the Cl hemisphere at 1 d, 7 d and 14 d after HI; they represent respectively ~48%, 19% and 9% of the injured hemisphere’s total CD11b + cell population. - The temporal pattern of peripheral leukocyte influx is biphasic, i..e it is characterized by peak accumulation of inflammatory cells (inflammatory monocytes and granulocytes) at 1 d, relative quiescence at 3 d and renewed infiltration at 7 d post-HI. *Immunohistochemistry with EGFP and Iba1* - At 1d post-HI, infiltration is disperse and observed in the hippocampus, thalamus and striatum, while at 7 d post-HI, the infiltration is dense and limited to hippocampus and white matter in the thalamus; cortical infiltration is observed at both time points but only in the case of severe HI injury. *Cytokines* - Inflammatory cytokine levels (e.g. IL-1α, IL-1β and IL-6) are elevated in the Il hemisphere in comparison to the Cl hemisphere at 6 h, 1, 3 and 14 d post-HI, but not at the 7 d time point. - Chemokine levels (MCP1, MIP1a) are elevated in the Il hemisphere in comparison to the Cl hemisphere, are reduced to sham levels within 1–3 and remain so for 2 w post-HI, except for KC which increases again markedly at 14 d post-HI. *Depletion of myeloid cells* via *repeated, systemic administration of an antibody RB6-8C5* Antibody treatment expectedly diminishes myeloid cell infiltration after neonatal HI; 14 d post-HI, the neuronal injury is reduced only in males, but not in females. The antibody treatment does not improve the loss in myelinated areas after HI in either sex.	- The infiltration of inflammatory monocytes and granulocytes after neonatal HI follows a temporal biphasic pattern, with the highest levels of invading cells at 1 d post-HI and a second wave of invasion occurring 7 d post-HI. - Antibody mediated depletion of myeloid cell infiltration after neonatal HI appears neuroprotective in males, but not in females.
Fisch U et al., 2020 PMID: 31954397	Sprague-Dawley rat P7 Right CCA ligation 40 min 8% O_2_ Sacrifice time-points: 3, 13 and 33 d	*Immunohistochemistry* - In comparison to sham, the density of Iba1^+^ microglia in the HI-Il SVZ remains elevated at P10, P20 and P40. - The proportion of activated microglia (Iba1^+^CD68^+^/Iba1^+^) in the Il SVZ is significantly increased at P10 and P20. - The proportion of proliferating microglia (Iba1^+^BrdU^+^/Iba1^+^) is augmented in the Il SVZ only at P10. - Morphologically, the proportion of amoeboid microglia in the Il SVZ rises significantly at P10, but not thereafter. Nevertheless, the number of ball-and-chain buds detected in Iba1^+^ microglia is higher at P40 in the Il SVZ than in the sham SVZ. *Microarray analyses of Cd11b*^*+*^ *microglia micro-dissected from the SVZ* - After neonatal HI, both pro- and anti-inflammatory related genes as well as neurotrophic genes (Igf-1) are upregulated at P10 and P20. - KEGG pathway analysis of differentially expressed genes of HI and sham SVZ microglia reveals an enrichment of pathways associated with neurodegenerative diseases in P10-HI SVZ microglia.	- Neonatal HI induces significant changes in the density, proliferation status and morphology of microglia in the SVZ for a prolonged period of time, and these parameters are less affected in adjacent regions i.e. cortex and corpus callosum. - Overall there is a SVZ-specific response of microglia to neonatal HI, associated with a complex immunomodulatory and potential neurotrophic gene expression pattern.

All sacrifice time-points are given in post-HI

This reinforces the idea that the M1/M2 concept, as currently applied to microglia should be questioned, if not abandoned [104, 105]. With the advent of RNA-sequencing (RNA-Seq) analyses of microglia, an extraordinary complexity and unique microglial signature during physiological development has been revealed. Thus, mouse microglia harvested between embryonic day E14.5 and P9 harbor a transcriptomic signature distinct from earlier embryonic stages or adult microglia, and these so called “pre-microglia” express of a common cluster of genes involved in neural migration, neurogenesis and cytokine secretion [106]. Further refining these observations, single-cell RNA-sequencing (scRNA-Seq) of murine microglia at E14.5 and P5 reveals a greater diversity of microglial subpopulations at these ages than at juvenile, adult or old stages [107]. Collectively these data highlight the peculiarity of the microglial phenotype during development, the regional and temporal specificities, and agrees with the reported heterogenous functions in the developing brain.

### Does Age at Time of Injury Influence the Ischemic-Induced Microglial Response? If So, how?

Six studies have directly compared the cerebral immune response after induction of HI at different postnatal ages, out of which three were conducted by the same laboratory (see Table 2). In these reports, the intensity of the microglial and cytokine response after neonatal HI (P9 mice or P12 rats) was compared to that after HI in newborn rats (P1), juvenile mice (P21 or P30) or adult mice (three months of age). While HI-induced microglia proliferation (five weeks post-HI) and brain expression of IL-18 and MCP-1 (three days post-HI) appeared more pronounced in the ipsilateral hippocampus of mice subjected to HI at P21 than in mice subjected to HI at P9, opposite results were documented in studies comparing neonatal HI to older juvenile or adult HI. In particular HI-associated elevation in hippocampal microglial population (CD11b^+high^/CD45^+low^) and in pro-inflammatory cytokines (TNF-α, IL-1β) two days after lesioning was significantly more robust in P9 than in P30 mice. Morphological analyses of microglia at the same time point post-HI corroborated these findings, with microglia displaying a more activated phenotype in P9 than in P30 mice. The acute neuroinflammatory response (48 h) after HI was also much stronger in P12 rats than in rats subjected to HI at P1 [79]. Using Cx3cr1^GFP/+^ Ccr2^RFP/+^ double transgenic mice to allow differential labeling of resident microglia (GFP^+^) and blood derived macrophages (RFP^+^), Umekawa et al. [14] showed that the increase in the total number of hippocampal microglia after HI was approximately the same in P9 and adult mice, but (i) it occurred later in adult mice and (ii) the proportion of activated microglia (GFP and galectin-3 double positive) was much higher in the immature than in the adult brain. In addition, one day after HI, the concentration of the pro-inflammatory chemokine CCL2 was around three times higher in the ipsilateral hippocampus of the P9 neonates than in the adult mice. Altogether the majority of the studies indicate that the intensity of the microglial response after HI is stronger in the immature rodent brain equivalent to a term-like human brain than in a less (P1 or preterm-like human brain) or more developed juvenile or adult brain. While the time course of HI-induced microglial activation is similar in neonatal and P30 brain, it seems delayed in the adult brain in comparison to the neonatal brain. These age-related differences may also impact response to treatment, as suggested by report of Cikla et al. [108] and detailed in next section. Overall, it is very likely that the microglial heterogeneity at the transcriptional level described during development [103] contributes to this differential response to HI and treatment, but exactly which signaling pathway(s) is (are) involved remain to be investigated.

**Table 2:** Impact of age on ischemic-induced neuroinflammation-Comparative studies

Reference	Species and Model of HI	Immune-related findings in the ipsilateral (Il) brain in comparison to the contralateral (Cl) brain	Conclusion(s)
Qiu L et al., 2007 PMID: 16926844	C57/BL6 male mice • P9 “P9 immature mice/brain“ Left CCA ligation 35 min 10% O_2_ • P21 “P21 juvenile mice/brain“ Left CCA ligation 30 min 10% O_2_ Sacrifice time points: 5 w for BrdU studies (i.e. P45 for P9 mice, and P57 for P21 mice) 3 d for IL-18 and MCP-1	*Immunohistochemistry in the hippocampus only (dentate gyrus = DG and Cornu Ammonis = CA)* - In the brain of sham-exposed animals, the total number of BrdU-labeled cells is greater in the immature than in the juvenile mice. Nevertheless, 5 w after HI, a greater increase in the number of BrdU^+^ cells is measured in the brain from juvenile mice than from immature mice. - In the CA: the number of BrdU^+^/Iba1^+^ cells decreases significantly during normal brain development, does not increase after HI at P9, but increases 113-fold after HI at P21. - In the DG: the number of BrdU^+^/Iba1^+^ cells does not change significantly during normal brain development or after HI at P9, but increases 8-fold after HI at P21. - The number of MCP-1 and IL-18 immunopositive cells in the CA and DG is twice higher in the Il hemisphere of mice subjected to HI at P21 than in that of mice subjected to HI at P9.	The post-ischemic hippocampal inflammation evaluated in terms of microglia proliferation and expression of IL-18 and MCP-1 is more pronounced in the hippocampus from mice subjected to HI at the juvenile stage than from mice subjected to HI at the neonatal stage.
Ferrazzano P et al., 2013 PMID: 23469850	C57BL/6 J mice • P9 “P9 neonatal mice/brain“ Left CCA ligation 50 min 10% O_2_ Sacrifice time points: P10, P11, P12, P16, P18, P21 and P26 • P30 “P30 juvenile mice/brain“ Left CCA ligation 50 min 10% O_2_ Sacrifice time points: P32, P39 and P47	*Flow cytometry* Mice subjected to HI at P9: Ipsilateral hippocampus, CD11b^+high^/CD45^+low^ population: - Large increase at 1 d post-HI, peak at 2 d post-HI, then gradual decrease to a low level by 1 w post-HI. This low level is sustained up to 17 d post-HI. - Marked increase in CD11b^+^/CD45^+medium^ population with no increase in CD11b^+^/CD45^+high^ population 2 d post-HI, suggesting absence of invasion of peripheral monocytes. Ipsilateral cortex, CD11b^+high^/CD45^+low^ population: - Onset time of the elevation in microglia/macrophage counts is delayed until 3 d post-HI, is muted compared to hippocampus (~55% of peak hippocampal counts), and is sustained for 12 d post-HI. By d 17 post-HI, the CD11b^+^/CD45^+^ counts are restored to a low level in the Il cortex. Ipsilateral striatum, CD11b^+high^/CD45^+low^ population: - Delayed onset time at 4 d post-HI and peak elevation at 12 d post-HI. In ipsilateral cortex and striatum, small population of CD11b^+^/CD45^+high^ 9 d post-HI, suggesting late invasion of peripheral macrophages.	- In the neonatal P9 brain, the microglial response in the hippocampus occurs earlier (peak on d 2 post-HI) than in the striatum and cortex (peak on d 9 post-HI). - The microglial proliferation and pro-inflammatory cytokine responses in P9 brains after HI are more robust in comparison to P30 brains. - Such differential inflammatory response induced by HI at the two postnatal stages may have implications for tissue repair.
Ferrazzano P et al., 2013 PMID: 23469850		Mice subjected to HI at P30: - Overall smaller increases in microglia/macrophage counts while similar regional time-course of microglia responses is observed. - Ipsilateral hippocampus: transient elevation of CD11b^+^/CD45^+^ population at 2 d post-HI. - Ipsilateral cortex and striatum: delayed increase in microglia response, CD11b^+^/CD45^+^ peak at 9 d post-HI and sustained increase until 17 d post-HI. - No CD11b^+^/CD45^+high^ cells in any region at any time point post-HI. *Cytokines* Mice subjected to HI at P9: - Ipsilateral hippocampus: transient elevation of TNF-α, IL-1β at 2 d post-HI, then return to the contralateral levels at 9 d post-HI; elevation of IL-10 at 2 d up until 9 d post-HI. - Ipsilateral cortex: delayed expression of TNF-α and IL1-β at d 9 post-HI. Mice subjected to HI at P30: - Smaller rise in TNF-α and IL-1β at 2 d post-HI than that in the P9 hippocampus. - The ipsilateral P30 cortex and striatum also show a smaller increase in TNF-α and IL-1β expression. - IL-10 levels are elevated at 2 d post-HI in P30 hippocampus at levels similar to the P9 hippocampus, and remain elevated at 9 d post-HI. - In P30 cortex and striatum, IL-10 levels are increased at 9 d post-HI.	
Umekawa T et al., 2015 PMID: 26179283	Cx3cr1^GFP/+^Ccr2^RFP/+^ double transgenic mice. Cx3cr1^GFP/+^: marking of resident microglia Ccr2^RFP/+^: marking of blood-derived macrophages • P9 Right CCA ligation 50 min 10% O_2_ • Adult (3 months of age or 3mo) Right CCA ligation 75 min 10% O_2_ (to produce similar extent of brain injury) Sacrifice time points: 1, 3, 7 and 28 d	- No significant differences in mortality rates or neuropathological scores between P9 and 3mo mice at any time point after HI. *Immunohistochemistry in the hippocampus only* - The Cx3cr1-GFP^+^ cells increase 1 d after HI in the DG and the CA regions of the P9 pups, whereas they increase 7 d after HI in the CA region of the 3mo mice. - 1 d post-HI, Ccr2-RFP^+^ cells appear in the hippocampus of the P9 pups, whereas they are very few in the 3mo mice. Then, they increase in the DG and the CA regions, peak 3 d after HI in both the P9 neonates and the 3mo mice, and then decrease gradually. - GFP^+^/galectin-3^+^ cells (activated microglia) are increased 1 and 3 d after HI in the P9 pups, whereas they reach a peak 3 and 7 d after HI in the 3mo mice. The number of GFP^+^/galectin-3^+^ cells is nine time greater in the immature than in the adult hippocampi. - RFP^+^/galectin-3^+^ cells (activated macrophages) in the DG and the CA regions increase transiently 3 d after HI in both the P9 pups and the 3mo mice. - The number of Cx3cr1-GFP^+^/CD16/32^+^ “pro-inflammatory-M1 “cells briefly increases 3 d after HI in the P9 hippocampus, whereas in the 3mo mice, these cells increase at 3 d and remain at the same level for up to 7 d after HI. - The number of Cx3cr1-GFP^+^/CD206^+^ “anti-inflammatory-M2 “cells increase transiently 3 d after HI in the DG region of both the P9 pups and the 3mo mice. - The number of RFP^+^/CD16/32^+^ in the DG increase 3 d after HI in both the P9 pups and the 3mo mice; same observation for the number of RFP^+^/CD206^+^ cells. *ELISA* - The concentration of CCL2 (MCP-1) at 1 d post-HI is 3 times higher in the ipsilateral hippocampus of the P9 pups compared with the 3mo mice.	- First report that demonstrates age-dependent differences in the activation of resident microglia and infiltrating blood-derived macrophages in the hippocampus after HI brain injury. - The inflammatory response appears more pronounced in the immature brain. - Overall, the resident microglia, rather than infiltrating blood-derived macrophages, proliferate and are activated earlier in the immature than in the adult hippocampus, but remain increased longer in the adult brain.
Cikla U et al., 2016 PMID: 26857490	C57BL/6 J mice • P9 “P9 neonatal mice/hippocampus“ • P30 “P30 juvenile mice/hippocampus“ Left CCA ligation 50 min 10% O_2_ Sacrifice time points: 2 and 9 d for brain analyses and 60 d for behavior assessment	*Flow cytometry* - 2 d after HI, robust increase in CD11b^+^/CD45^+^ microglia counts (ratio Il/Cl) in P9 (~ 9.5 fold) and in P30 hippocampus, although less pronounced (~ 5.7 fold); modest increase in microglia counts in cortex and striatum. - 9 d after HI, decline in microglia counts in hippocampus, and dramatic increase in microglia counts in cortex and striatum in both P9 and P30 mice. - Minocycline reduces microglia counts in P9 mice at 2 and 9 d post-HI in all regions, but is ineffective at reducing microglia counts 9 d post-HI in P30 cortex and striatum. *Iba1 immunohistochemistry* - In the hippocampus, with minocycline treatment, microglia retain a ramified morphology at 2 d post-HI in both P9 and P30 mice. - In the cortex and striatum of minocycline-treated P9 mice, microglia demonstrate a ramified morphology in the cortex and striatum at d 9 post-HI. - However, in minocycline-treated P30 mice, microglia in Il cortex and striatum display activated morphology similar to that seen in the vehicle-treated controls at d 9 post-HI.	- Despite early improvement in neurological damage (assessed by MAP2 immunohistochemical staining) and in microglia parameters in minocycline-treated P9 mice, no improvement in cerebral atrophy (measured by MRI) or hippocampus learning behavior evaluated 60 d post-HI are detected. - Conversely, minocycline does not reduce neurological damage in P30 HI-exposed mice; yet 60 d post-HI, the treatment results in less cerebral atrophy and is accompanied by improved hippocampal learning. - Minocycline has a differential impact depending on the age at which HI is inflicted. The mechanisms remain unclear but late microglial activation could have a beneficial role.
Cengiz P et al., 2019 PMID:30639264	C57BL/6 J mice • P9 “P9 neonatal mice/hippocampus“ • P30 “P30 juvenile mice/hippocampus“ Left CCA ligation 50 min 10% O_2_ Sacrifice time points: 2 d	*Iba1 immunohistochemistry in the Il hippocampus (in comparison to Cl hippocampus)* - In mice subjected to HI at P9, microglia demonstrate a significant decrease in branching measures and an increase in cell body volume, reflecting intense microglia activation. - In mice subjected to HI at P30, microglia show a much more moderate decrease in branching morphology 2 d post-HI, and overall retain a highly ramified morphology. *Gene expression in CD11b*^*+*^ *microglia isolated from hippocampus* - In mice subjected to HI at P9, a significant reduction in TGF-β receptor expression, but an increase in Serpine1 (part of the TGF-β receptor signaling pathway) expression are observed. Also, there is a significant reduction in MerTK (Mer Tyrosine Kinase, phagocytic receptor) and SOCS3 (suppressor of cytokine signaling, component of the MerTK pathway) expression. - In mice subjected to HI at P30, the expression of TGF-β receptor is also significantly reduced; Serpine1 expression is not changed 2 d after HI, but its expression remains dramatically elevated compared to P9. As in P9 mice after HI, there is a reduction in MerTK and SOCS3 expression; but the expression of both is significantly higher in the Il P30 compared to Il P9 hippocampus.	- During physiological development (between P2 and P60), microglia in the hippocampus undergo significant changes in morphology, reaching a mature phenotype by P30. These morphological changes are accompanied by increases in the expression of members of the TGF-β receptor and MerTK signaling pathways. - Based on morphological evaluation, 2 d after HI, there is an intense activation of microglia in the Il hippocampus of P9 mice, but not in the P30 mice. - The age-related differences in the microglial response to neonatal HI may be linked in part to the age-associated changes in the TGF-β receptor and MerTK signaling pathways.

All sacrifice time-points are given in post-HI

Box 3 the Fundamental Breakthroughs in Immunology that Led to the M1/M2 Concept

**Table:** 

Mackaness, in his fundamental work between 1962-1970 [109–112] (for an in-depth explanation of his work, see Van Epps [113]) introduced first the term “activated macrophage”. Using a mouse model of infection with the intracellular bacteria *Listeria monocytogenes*, he demonstrated that the mechanism of “acquired cellular resistance”— the fact that after a primary infection, mice become temporally resistant to a subsequent reinfection with the same or unrelated intracellular pathogen— was dependent upon a change into the host macrophages that became “activated”. He further showed that macrophage activation depended on a soluble factor released by lymphocytes—now recognized as a specific subset of T helper CD4^+^ cells—exposed to an antigen [111]. He defined the activated macrophage as “larger and much more complex morphologically; it has a marked propensity to spread on glass, a property which is related to its enhanced capacity for phagocytosis. The content of acid hydrolases, the digestive capacity, the respiratory rate and the mitotic rate of activated macrophages are all conspicuously raised.” [112].	
The major cytokine responsible for the observed macrophage activation (now referred to as “classical” activation) was thereafter identified as IFN-γ [114]. Further studies on the effect of different cytokines on macrophage activation showed that IL-4 [115] and IL-13 [116] caused a macrophage phenotype distinct from that induced by IFN-γ. This “alternative” activation was characterized, among others, by a downregulation of IFN-γ mediated bactericide signals such as NO production, a reduction in the release of inflammatory cytokines, and an increased expression of an endocytic receptor, namely mannose receptor (or CD206).	
Amidst the work on macrophage activation, a major step in the understanding of immune regulation was brought about by the T helper type 1 (TH1)-TH2 hypothesis, proposed by Coffman & colleagues in 1986 [117, 118]. Further insight into the model and its historical context can be found in [119, 120]. In essence, it provided an invaluable framework to describe how an organism responds to different pathogens (e.g. intracellular vs extracellular). The TH1 and TH2 clones were distinguished by the pattern of cytokine release, with TH1 clones secreting mainly IFN-γ, and TH2 clones IL-4, IL-5, and IL-13. In general, TH1 and TH2 responses are associated with cellular and humoral immunity, respectively.	

Box 4 the M1/M2 Concept of Macrophage/Microglia Activation

**Table:** 

Mills in 2000 applied the TH1/ TH2 nomenclature to define macrophage activation, hence proposing the concept of M1/M2 macrophage activation [121]. The M1/M2 model stemmed from the main following in vitro data: (i) macrophages from C57BL/6 and Balb/c mice, described to be prototypical Th1 and Th2 strains respectively, respond from a metabolic point of view, i.e. in terms of NO and ornithine production, differently to the same Th1-like stimuli, namely IFN-γ, LPS, or IFN-γ + LPS. In particular, while stimulated macrophages from C57BL/6 produce predominantly NO (iNOS or kill pathway), macrophages from Balb/c produce predominantly ornithine/urea (arginase or Heal pathway). (ii) The same observation was made for macrophages isolated from SCID or NUDE C57BL/6 or Balb/c mice, in which lymphocytes are reduced or absent. This led Mills to hypothesize that macrophages have an inherent capability to display a classical M1 or alternative M2 phenotype, and do not necessarily need “instruction” from lymphocytes. He further proposed that macrophages are in fact the first line of defense cells that can then direct the nature of the adaptive immune response of the T lymphocytes, i.e. TH1, TH2 or other [122–124]. This latter view remains nevertheless contested by immunologists who consider the lymphocytes and the cytokines they release as the orchestrators of macrophage activation [125]. A notable difference between the Th1/Th2 and the M1/M2 hypotheses is that M1/M2 polarized macrophages do not constitute separate clones, like Th1/Th2 cells, but rather represent the extremes of a spectrum of different phenotypes depending on stimulus/environment.	
The M1/M2 nomenclature was then enlarged, with the introduction of M2a, M2b, M2c [126] and M2d subgroups [127, 128], defined by partially overlapping or distinct transcriptional profiles/signaling cascades induced by specific stimuli. Immunologists now suggest to use a more precise polarization nomenclature that includes the source of macrophage, the stimulus used and corresponding set of activation markers [125]. Thus, while the value of the M1/M2 framework is acknowledged, it is also recognized that it does not fully capture the complexity of macrophage responses especially in in vivo settings, as now revealed by the numerous analyses of macrophage transcriptomes via high-throughput RNA sequencing-bulk, single cell or single nucleus—methodologies in diverse pathological conditions (for reviews, see [129, 130]).	
Even though it may only seem natural that the M1/M2 concept was also applied to microglia, the brain resident macrophages, neuro/immunologists are reconsidering the use and the validity of such concept [104, 105, 131]. As for macrophages, microglial gene expression profiles typically cannot be fitted into M1- or M2-like subphenotypes, as observed in rat models of adult ischemic stroke [132] or neonatal HI [98], in rodent models of neurodegenerative disorders [100, 107, 133, 134] and in human microglia isolated from Alzheimer’s patients [135, 136]. There again, the complexity of microglia cannot be reflected into the M1/M2 concept.	

## Modulation of the Immune Response and Neuroprotection-Correlative or Causal Crosstalk?

Treatments known to confer some degree of neuroprotection can modulate aspects of HI-induced inflammation. For instance, hypothermia can reduce infarct size, improve behavioral outcome and these effects are concomitant with a decrease in cytokine expression in the injured hemisphere (IL-18, IL-6, TNF-α), and a reduction in the number of amoeboid microglia in cortex and corpus callosum [137–139]. But whether the direct modulation of the immune response associates with neuroprotection is a challenging question, because anti-inflammatory agents/treatments can have biological actions unrelated to inflammation, for instance on angiogenesis, cell proliferation and apoptosis. Such broad effects are documented for erythropoietin [140], cyclooxygenase-2 (COX-2) inhibitors [141], doxycycline and minocycline [142].

### General considerations.

Twelve immune-related therapies have been tested in the rodent model of neonatal HI (see Table 3). Among them, only EPO has been used in clinical trials for neonates with HIE (reviewed in [143]); seven are in clinical use for other human conditions (recombinant human interleukin-1 receptor antagonist, platelet-activating factor antagonists, minocycline, doxycycline, cromolyn, etanercept and fingolimod); one is going out of clinic due to cardiac side effects (COX-2 inhibitor); and three are for preclinical testing only (anti-rat neutrophil serum (ANS), IKK/NF-κB inhibitor and JNK inhibitor). Regardless of clinical use, testing in animal models remains invaluable because it provides fundamental knowledge on the molecular or cellular inflammatory components contributing to the pathogenesis of neonatal HI, and on the phase of injury post-HI these components may be involved in.

**Table 3:** Effects of immune-related treatments in the rodent model of neonatal HI

Reference	Species and Model of HI	Immune-related treatment	Degree of neuroprotection^†^, neuroinflammation, conclusion(s)
Martin D et al., 1994 PMID: 7867766	Sprague-Dawley rat P7 Left CCA ligation 120 min 7.5% O_2_ Sacrifice time-point: 7 and 14 d (P14 and P21)	**Recombinant human interleukin-1 receptor antagonist (rhIL-1ra, anakinra)** Route: Repeated subcutaneous (s.c.) administration Dose: 100 mg/kg Treatment protocol: **In the acute phase post-HI** 4 groups of rat neonates receiving different dosing frequencies prior to and/or after the HI insult (up till 8 s.c. injections) - Group 1: 1 h prior hypoxia and at 0, 1, 3, 5, 9, 20 and 28 h - Group 2: at onset of hypoxia and at 1, 3, 5, 9, 20 and 28 h - Group 3: 1 h post hypoxia, and then 3, 5, 9, 20 and 28 h - Group 4: 3 h post hypoxia, and then 5, 7, 20 and 28 h In clinical use for other conditions: yes, e.g. for gout	*Degree of neuroprotection* 7 d post-HI, assessment of right and left hemisphere dry weights disparities: - Group 1: ~55% neuroprotection (*). - Group 2: ~95% neuroprotection (**). - Group 3: ~80% neuroprotection (**). - Group 4: ~54% neuroprotection (NS). 14 d post-HI, evaluation of loss of striatal neurons in cresyl violet stained brain slices only in group 1: - ~65% neuroprotection (*). *Cerebral inflammation post-treatment* Not examined *Conclusion* IL-1 receptor activation plays a role in the pathogenesis of neonatal HI brain injury.
Hagberg H et al., 1996 PMID: 8888290	Wistar rat P7 Left CCA ligation 70–100 min 7.70% O_2_ Sacrifice time-points: 1, 3, 6, 10, 20, and 48 h and 14 d (P21)	**rhIL-1ra** Route: Intracerebral administration in the ipsilateral hemisphere Dose: 3.3 or 5 μg/rat pup Treatment protocol: **Prophylactic, or in the acute phase post-HI** Administration either before (3.3 μg/rat pup) or immediately after HI (5 μg/rat pup) In clinical use for other conditions: see above	*Degree of neuroprotection* 14 d post-HI, assessment of right and left hemisphere dry weights disparities: - HI duration of 100 min and IL-1ra pretreatment: ~24% neuroprotection (**) - HI duration of 70 min and IL-1ra post-treatment: no change in mean injury *Cerebral inflammation post-treatment* Not examined *Conclusion* rhIL-1ra mediated neuroprotection, despite being moderate, suggests the pathophysiological significance of IL-1 in neonatal HI.
Liu X et al., 1996 PMID: 8947953	Sprague-Dawley rat P7 Right CCA ligation 150–195 min 8% O_2_ Sacrifice time-point: 5 d (P12)	**Platelet-activating factor (PAF) antagonist BN52021** Route: intraperitoneal (i.p.) injection 3 treatment protocols: **Prophylactic, or in the acute phase post-HI** - Pretreatment: 2 serial injections (25 mg/kg/injection), 1 immediately before the onset of hypoxia, and a second one 1 h after the end of hypoxia - Post-treatment: 2 serial injections (25 mg/kg/injection), 1 immediately after the end of hypoxia, and then 2 h later - Post-treatment-dose effect: 2 injections, immediately and 2 h after the end of hypoxia- different doses tested (12.5, 25 or 50 mg/kg/injection) In clinical use for other conditions: PAF antagonists are a heterogenous group of medication, some of which are used for different diseases	*Degree of neuroprotection* 5 d post-HI, assessed on cresyl violet stained brain slices: - Incidence of infarction 90% in vehicle-treated rats vs 33% in BN52021-pretreated rats (**) and 30% in BN52021-post-treated rats (*). - Neuroprotection per region • Pretreatment: 82% in cortex, 74% in striatum, 90% in hippocampus. • Post-treatment: 72% in cortex, 46% in striatum, 73% in hippocampus. 5 d post-HI, assessed by interhemispheric weight differences for the post-treatment dose-effect study: 18.9% (NS), 44.8% (*) and 32.5% (*) neuroprotection observed in rats treated with 12.5, 25 or 50 mg/kg/dose compared with controls; differences in neuroprotection among the three doses not statistically different. *Cerebral inflammation post-treatment* Not examined *Conclusion* PAF appears to be one of the acute inflammatory factor mediating perinatal HI brain injury; PAF antagonists may represent a pharmacologic approach to attenuate the severity of HI-brain injury.
Hudome S et al., 1997 PMID: 9128280	Wistar rat P7 Right CCA ligation 135 min 8% O_2_ Sacrifice time-points: 0, 5, and 30 min, and 2, 4, 8, 18, and 42 h	**Anti-rat neutrophil serum (ANS) compared to allopurinol** Note: rabbit polyclonal anti-rat neutrophil serum (ANS) is used to deplete neutrophils; comparison to allopurinol, a drug that reduces neutrophil accumulation, and a known neuroprotective agent Route: i.p. for ANS and s.c. for allopurinol Dose: Allopurinol 135 mg/kg Treatment protocol: **In the acute phase post-HI** - 4 treatment groups: allopurinol, neutropenic, both and none - ANS is injected i.p. at time of surgery - Allopurinol is injected 15 min post-hypoxia In clinical use for other conditions: no, ANS is experimental only	*Degree of neuroprotection* 42 h post-HI, assessed by quantifying brain swelling (wet vs dry weight of posterolateral half of each cerebral hemisphere): - ~70% (**) reduction in brain swelling by ANS-induced neutropenia and allopurinol. The application of both treatments confers no synergistic effect. *Cerebral inflammation post-treatment* Not examined. *Conclusion by study authors* Neutropenia before the insult is neuroprotective, thus neutrophils may play a role in the pathogenesis of neonatal HI, although the mechanisms remain unclear.
Arvin K et al., 2002 PMID: 12112047	Sprague-Dawley rat P7 Left CCA ligation 150 min 8% O_2_ Sacrifice time-points: 24 h and 1 w	**Minocycline** Route: i.p. Dose: 45 mg/kg Treatment protocol: **Prophylactic, or in the acute phase post-HI** Single injection immediately before placement in hypoxic chamber, or immediately after removal from hypoxic chamber In clinical use for other conditions: yes, for various infections	*Degree of neuroprotection* 7 d post-HI, measurement of the amount of surviving tissue in cresyl violet stained brain slices, and calculation of % tissue loss in HI-IL vs HI-CL hemisphere: - Minocycline 45 mg/kg before hypoxia: 72% (****), 100% (***) and 85% (****) protection in striatum, hippocampus, and cortex. - Minocycline 22.5 mg/kg before hypoxia: 61% (NS), 72% (NS) and 71% (*) protection in striatum, hippocampus, and cortex. - Minocycline 45 mg/kg immediately after hypoxia: similar results as when given before hypoxia. - Minocycline 45 mg/kg 3 h after hypoxia: 28% (NS), 9% (NS) and 22% (NS) protection in striatum, hippocampus, and cortex. *Cerebral inflammation post-treatment* Not examined *Conclusion* Minocycline at 45 mg/kg provides near complete neuroprotection after neonatal HI only if given immediately before or after HI. Administration 3 h post-HI is not neuroprotective.
Palmer C et al., 2004 PMID: 14739365	Wistar rat P7 Right CCA ligation 135 min 8% O_2_ Sacrifice time-points: - 42 h post-HI for brain swelling - 14 d post-HI for brain atrophy - 0 and 6 h post-HI for analysis of brain energy metabolites	**Anti-rat neutrophil serum (ANS)** Route: s.c. Treatment protocol: **Prophylactic, or in the acute phase post-HI** - before HI group: immediately after carotid ligation - after HI group: 4–8 h after hypoxia - 2 corresponding control groups (HI animals exposed to normal rabbit serum) In clinical use for other conditions: no, experimental only	*Degree of neuroprotection* 42 h post-HI, measurement of brain swelling: - In the before HI group, neutropenia reduces brain swelling by 75% (***). - In the after HI group, no reduction in brain swelling. - 14 d post-HI, measurement of brain atrophy: - In the before HI group, neutropenia reduces IL hemisphere atrophy by 61% (*). - In the after HI group, neutropenia is not protective. *Cerebral inflammation post-treatment* Not examined *Conclusion* Neutrophils play a role in early HI-induced neuropathogenesis. Anti-neutrophil strategies should be applied very early after HI to be neuroprotective.
Jantzie L et al., 2005 PMID: 15647741	Sprague-Dawley rat P7 Right CCA ligation 150 min 8% O_2_ Sacrifice time-points: - 1 w (P14) for brain stainings - 1, 2, and 4 h for immunoblotting	**Doxycycline (DOXY)** Route: i.p. Dose: 10 mg/kg Treatment protocol: **Prophylactic, or in the acute phase post-HI** Injection of DOXY as a one-time dose - Immediately before HI - 1 h after HI - 2 h after HI - 3 h after HI In clinical use for other conditions: yes, for various infections Can cross BBB	*Degree of neuroprotection* 7 d post-HI, assessment of NeuN immunoreactivity: In CA1 hippocampus, the average cell loss is ~30% in control animals, whereas it is ~10% in animals treated with DOXY, regardless of injection time-points, which means a ~ 70% neuroprotection. *Cerebral inflammation post-treatment* At 7 d post-HI, trend towards a reduction in the number of ED-1^+^ cells in the IL hippocampus, thalamus and cortex, with a greater extent when DOXY is administered before HI. *Conclusion* DOXY protects the neonatal brain from HI when applied in a short time window post-HI.
Sun Y et al., 2005 PMID: 16040592	Sprague-Dawley rat P7 Right CCA ligation 150 min 8% O_2_ Sacrifice time-points: 3, 7, 14, and 21 d	**recombinant human Erythropoietin (rh-EPO)**^**§**^ Route: i.p. Dose: 5 U/g body weight Treatment protocol: **In the acute and subacute phase post-HI** 3 injections, namely injection at 24 h post-HI and for 2 additional days In clinical use for other conditions: yes, and in clinical trials for infants with CP (NCT# 02811263) Can cross a dysfunctional blood-brain barrier	*Degree of neuroprotection* 7, 14 and 21 d post-HI, assessed by measurement of IL and CL wet weight disparities: ~47–77% (***) protection at these 3 time-points. *Cerebral inflammation post-treatment* - rh-EPO abolishes HI-induced rise in IL-1β protein levels in the ipsilateral hemisphere 3, 7, and 14 d post-injury, but does not affect mRNA levels of TNF-α between 3 and 14 d post-HI. - It limits HI-induced leukocyte infiltration in the parieto-occipital cortex, observed with CD4 and CD68 IHC stainings. *Conclusion* 3 doses of rh-EPO is neuroprotective after HI. A potential mechanism of neuroprotection may be through reducing HI-associated elevation in IL-1β and through limiting leukocyte infiltration.
Nijboer C et al., 2008 PMID: 18420952	Wistar rat P7 Right CCA ligation 120 min 8% O_2_ Sacrifice time-points: 1 h, 3 h, 6 h, 12 h, 48 h and 6 w	**TAT-NBD, IKK/NF-κB inhibitor** - TAT-NBD: peptide inhibitor of the IKK complex, the NEMO Binding Domain (NBD)-peptide coupled to the protein transduction sequence of HIV-TAT to facilitate cerebral uptake - mutant TAT-NBD - biotinylated TAT-NBD Route: i.p. Dose: 20 mg/kg Treatment protocol: **In the acute phase post-HI** administration at various time points post-HI: 0, 3, 6, 9 and 12 h after HI In clinical use for other conditions: no specific NF-κB inhibitor in use yet, but unrelated drugs may target this signaling pathway Can cross BBB	*Degree of neuroprotection* 48 h and 6 w post-HI, assessed by MAP2 staining and hematoxylin-eosin respectively: - ~40–56% (**) protection at 48 h post-HI if TAT-NBD administered 0/3 h after HI, ~35% (*) if administered 6 h after HI. - ~75% (***) protection at 6 w post-HI if administered 0/3 h after HI. - Administration of TAT-NBD at 9 or 12 h post-HI is not neuroprotective. *Cerebral inflammation post-treatment* TAT-NBD treatment immediately after HI does not induce any changes in the HI-induced increase in mRNA levels of cytokine (TNF-α, IL-1β, IL-4, IL-10 and IL-1RA) at 3 h post-HI, even though NF-κB activity is fully inhibited at this time point. *Conclusion* Inhibition of the cerebral IKK/NF-kB pathway early after HI is neuroprotective, but does not involve abrogation of early cytokine expression. This early cytokine induction by neonatal HI may contribute to neuroprotection.
Jin Y et al., 2009 PMID: 19520991	Wistar rat P7 Right CCA ligation 75 min 8% O_2_ Sacrifice time-points: 0, 1 h, 2 h, 4 h, 24 h, 48 h and 4 w	**cromolyn, mast cell stabilizer** Route: s.c. Dose: 50 mg/kg body weight Treatment protocol: **In the acute phase post-HI** 3 injections, i.e. immediately after HI, and 1 and 24 h post-HI In clinical use for other conditions: yes, for asthma	*Degree of neuroprotection* 1, 2 and 4 w post-HI, scoring of Fluoro-Jade B stainings, and measurement of IL/CL hemisphere ratio on H&E stained brain slices: - 61-75% (*) improvement in score of brain damage at the time-points examined. - Cavitation is absent in all cromolyn treated rats, whereas it is present in 30% and 50% of vehicle-treated animals at 2 and 4 w post-HI, respectively. - IL/CL ratio restored to normal in cromolyn treated rats (*). *Cerebral inflammation post-treatment* Astrocytic and microglial activation, scored with GFAP and OX-42 antibodies respectively, are significantly reduced at the time points examined. *Conclusion* Mast cells appear to contribute to the inflammatory response to neonatal HI and could be the initiators of the immune response.
Nijboer C et al., 2009 PMID: 19628795	Wistar rat P7 Right CCA ligation 120 min 8% O_2_ Sacrifice time-points: 3, 6, 24 and 48 h	**TAT-NBD, IKK/NF-κB inhibitor; TAT-JBD, JNK inhibitor; and etanercept, TNF-α inhibitor** TAT-NBD, mutant TAT-NBD or TAT-JBD Route: i.p. Dose: 20 mg/kg for TAT-NBD peptides and 10 mg/kg for TAT-JBD Treatment protocol: administered 0 and 3 h after HI Etanercept (a fusion protein of TNF-R2 and human IgG_1_): Route: i.p. Dose: 5 mg/kg Treatment protocol: **In the acute phase post-HI** administered directly after HI alone or with TAT-NBD In clinical use for other conditions: etanercept is used for treating some autoimmune diseases (e.g. rheumatoid arthritis); the other compounds are experimental only	*Degree of neuroprotection* 48 h post-HI, measurement of CL and IL areas on MAP2 stained brain slices: - NBD: 75–88% (***). -JBD: 25% (*). - NBD + JBD: 50% (***). - Etanercept: 25% (*). - NBD + Etanercept: 44% (* vs NBD). *Cerebral inflammation post-treatment* - Combined NF-κB inhibition and JNK/AP-1 prevents HI-induced TNF-α production and reduces neuroprotection. - Etanercept alone has a significant neuroprotective effect, but not as great as that provided by NF-κB inhibition. - Etanercept treatment reduces the protective effect of TAT-NBD. *Conclusion* JNK/AP-1 inhibition and TNF-α inhibition reduce the neuroprotective effect of NF-κB inhibition: after HI is mediated in part by JNK/AP-1 activity and TNF-α production.
Fathali N et al., 2010 PMID: 20029340	Sprague-Dawley rat P10 Right CCA ligation 120 min 8% O_2_ Sacrifice time-points: 72 h, 2 w, or 6 w	**NS398, selective cyclooxygenase-2 (COX-2) inhibitor** Route: i.p. Dose: 2 dosage regimens, either 10 mg/kg (NS-10 group), or 30 mg/kg (NS-30 group) Treatment protocol: **In the acute and subacute phase post-HI** six injections (1, 6, 24, 36, 48, and 60 h) after hypoxia In clinical use for other conditions: going out of clinic due to adverse cardiac events	*Degree of neuroprotection* 2 and 6 w post-HI, measurement of IL/CL weight ratio: - NS-10 group: 63% at 2 w (*) and 57% (*) at 6 w. - NS-30 group: 79% at 2 w (*) and 75% (*) at 6 w. 6 w post-HI, behavior analysis: improvement in, or restoration of many HI-induced neurobehavioral deficits in NS-10 and NS-30 treated animals. *Cerebral inflammation post-treatment* - COX-2 immunopositive signals (western blotting and IHC staining of ipsilateral brain) are significantly reduced in NS398 treated HI animals in comparison to HI-vehicle animals. - At 72 h post-HI, IL-6 staining and concentration in ipsilateral brain is increased in HI-vehicle exposed rats, and significantly reduced by NS-30. Qualitative evaluation of single immunofluorescent staining for Iba1, CD68 and MPO in ipsilateral brain indicates that NS30 attenuates significantly HI-induced increase in these markers. *Conclusion* COX-2 inhibition is neuroprotective; effects may be mediated by anti-inflammatory actions, through IL-6 reduction, decreased activation of microglia and of infiltration of macrophages and neutrophils.
Cikla U, 2016 PMID: 26857490	C57BL/6 J mice - P9 “P9 neonatal mice/brain” - P30 “P30 juvenile mice/brain” Left CCA ligation 50 min 10% O_2_ Sacrifice time-points: 2, 9 and 60 d	**Minocycline** Route: i.p. Dose: 40 mg/kg Treatment protocol: **In the acute phase post-HI** Two injections, one at 2 h and the second one at 24 h post-HI In clinical use for other conditions: see above	*Degree of neuroprotection* 2 and 9 d post-HI, scoring of damage on MAP2 stained brain slices: - P9 neonatal brain: 73% (*) and 87% (*) protection at 2 and 9 d post-HI. - P30 juvenile brain: no protection at both days; of note, HI-induced brain damage less severe at P30 than at P9. Brain atrophy at 9 and 60 d post-HI by T2-weighted MRI: - P9 neonatal brain: no significant impact on volume loss at both days. - P30 juvenile brain: no significant impact on volume loss at 9 d, but a slight impact at 60 d. Hippocampal learning by Morris water maze test: - P9 neonatal brain: no amelioration in HI-induced learning deficits. - P30 juvenile brain: significant improvement. *Cerebral inflammation post-treatment* Impact of minocycline on total counts of microglia (CD11b^+^/CD45^+^) - Significantly suppresses HI-induced increase in microglia counts in the IL hippocampus from P9 and P30 brains 2 and 9 d post-HI; this is also observed in the cortex and striatum from P9 brains, but less in the same regions from P30 brains. Impact of minocycline on percentage of activated microglia (CD45^+med^/total microglia) - 2 d post-HI, in the IL hippocampus, induces ~70% reduction in the proportion of activated microglia in both neonatal and juvenile brains. - 9 d post-HI in the IL cortex and striatum - P9 neonatal brain: significant decrease in the percentage of activated microglia - P30 juvenile brain: no impact of minocycline on HI-induced late microglia activation *Conclusion* Minocycline has different neurological outcome depending on the age at which HI-injury is induced. Early improvements in neurologic injury do not necessarily predict long-term improvements in neurologic function.
Herz J et al., 2018 PMID: 30127782	C57BL/6 J mice P9 Right CCA ligation 60 min 10% O_2_ Sacrifice time-points: 1 w (P16)	**FTY720 (Fingolimod), a sphingosin-1-phosphate analog that reduces peripheral lymphocytes** Route: i.p. Dose: 1 mg/kg body weight Treatment protocol: **In the acute phase post-HI** single injection within 20 min after hypoxia In clinical use for other conditions: yes, for multiple sclerosis	*Degree of neuroprotection* 1 w post-HI, scoring of injury in cresyl violet stained brain slices: - Significant worsening of neuropathology in FTY720-treated animals versus saline-exposed mice. *Cerebral inflammation post-treatment* - FTY720 treatment induces a slight but not significant increase in Iba1 protein levels (assessed by western blot). - Despite FTY720 induced depletion of circulating T cells, the total cerebral leukocyte infiltration assessed qualitatively by IHC is unaffected by FTY720. This is explained by an increased infiltration of innate immune cells, mainly neutrophils and inflammatory macrophages. *Conclusion* Pharmacological mediated T cell depletion increases HI-induced brain injury; this contrasts with the neuroprotective effect of FTY720 observed in adult models of stroke. Thus, neonatal T cells may promote endogenous neuroprotection.

All sacrifice time-points are given in post-HI

H&E: hematoxylin-eosin

BBB: Blood brain barrier

^†^ Percent neuroprotection was either reported by study authors, either calculated by reviewers using the formula 100*(1-%damage in treated animals/%damage in vehicle treated animals), as in [146]. The significance is that reported by authors, and unless indicated, represents the significance of HI drug-treated animals versus HI vehicle-treated animals. Significance values * *P* < 0.05; ** *P* < 0.01; *** *P* < 0.001; **** *P* < 0.0001

^§^ Various rhEPO treatment protocols have been tested in the P7 rodent model of neonatal HI (for a review, see [143]). This study is included in the table because it is the only one that reports on CD68 immunostaining in the ipsilateral hemisphere from HI-exposed animals. The study authors attribute CD68 staining to infiltrating leukocytes, but it could also be microglia/macrophages

**Fig. 1:**
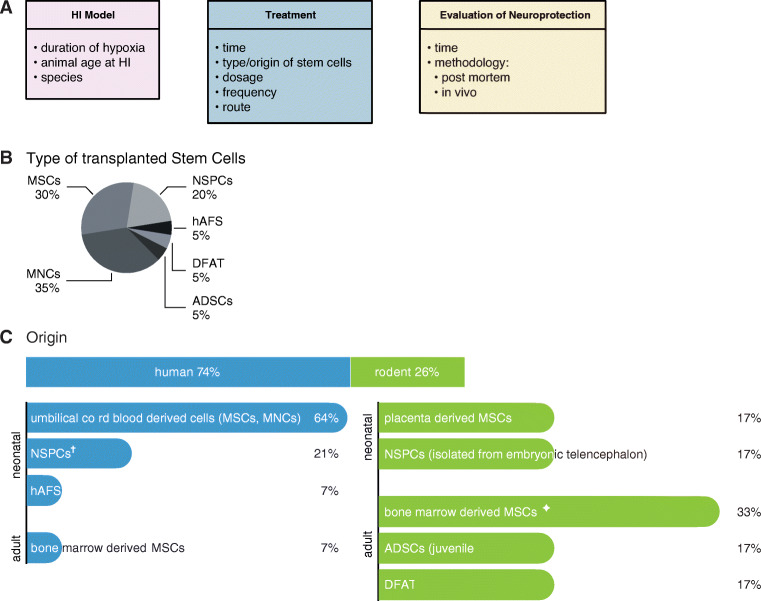
(**A**) Main experimental steps within which individual factors can eventually complicate interpretation of results between different studies. Charts displaying (**B**) type and (**C**) origin of transplanted SCs used in studies listed in Table 4

Eleven out of these twelve treatments conferred neuroprotection, but with highly variable degrees (see Table 3). Putative factors contributing to such variability for different or sometimes same treatments are listed in Fig. 1A. Studies in which minocycline was evaluated illustrate this point. In a rat model of neonatal HI, a single intraperitoneal dose of minocycline (45 mg/kg body weight BW) injected before or immediately after hypoxia induced a near complete neuroprotection at seven days post-HI. Nevertheless, if administered three hours post-HI, neuroprotection was almost lost. A lower dose (22.5 mg/kg BW) given before or immediately after hypoxia did not provide significant neuroprotection [144]. In a mouse model of neonatal HI, two intraperitoneal injections of minocycline (40 mg/kg BW) at two and 24 h after HI improved neurological injury two and nine days later, but the cerebral atrophy, measured with T2-weighed MRI nine and 60 days post-HI was similar to that of non-treated HI-exposed mice. Assessment of cognitive performance 60 days post-HI revealed no significant difference between treated and untreated HI mice. In that study, authors report that the same minocycline treatment protocol applied to juvenile mice subjected to HI at P30 ameliorated cognitive performance and cerebral atrophy measured 60 days after HI [108]. These two studies demonstrate how several parameters may ultimately influence response to treatment, namely (i) species, (ii) timing of administration, (iii) dosage, (iv) age at surgery, (v) time and methodology for evaluation of neuroprotection. Differing degrees of neuroprotection were also reported for recombinant human interleukin-1 receptor antagonist (rhIL-1ra, anakinra), namely >50% in Martin et al. [145] and only ~24% in Hagberg et al. [24]. Here the contributing factors to variability include the route of administration (subcutaneous-s.c. versus intracerebral-i.c.), the treatment protocol (multiple versus single injection), and dosage (multiple s.c. doses of ~1.5 g per rat in Hagberg’s report, versus a single intracerebral dose of 3.3 or 5 μg per rat in Martin’s study).

The role of dosage was documented for NS398, a COX-2 inhibitor for which a higher dose was better than a lower dose [146], while the platelet-activating factor antagonist BN 52021 exerted beneficial impact in a dose-independent manner [147].

A common feature between these studies is that all the immune-related therapies were either administered before hypoxia or within few hours after HI. Four out of five studies in which impact of treatment was tested prophylactically (before the onset of hypoxia, anakinra, PAF antagonist, minocycline, ANS) reported superior neuroprotective outcome in comparison to that with post-HI treatment, which could be of interest for at risk pregnancies. Of note, only one study reported worsening of infarct severity upon treatment (i.e., with Fingolimod, [21]). This potential bias towards publishing only beneficial outcomes may be counterproductive, as any “detrimental” treatment actually reveals the key involvement of the targeted pathway in mitigating the injury, a fact that could be harnessed further in therapies. Overall, based on these studies, the following cells and molecular pathways have been identified to play a role in the pathogenesis of neonatal HI in the acute phase: neutrophils, mast cells, platelet-activating factor, IKK/NF-κB signaling, and cyclooxygenase-2 since inhibiting them confer neuroprotection. No or a limited role in neuroprotection post-HI was reported for TNF-α and JNK pathway, while a beneficial role was documented for peripheral lymphocytes. The role of IL-1 signaling pathway remains currently unclear and requires further investigation.

**Is the microglial phenotype modulated by immune related treatments and does it correlate with neuroprotection?**

Among the 14 studies listed in Table 3, five examined the phenotype of microglia, but the depth of examination varied widely between studies. The data described below present results observed in the ipsilateral hemisphere from treated versus untreated HI-exposed animals. The injection of doxycycline before or within 3 h of HI reduced neuronal loss by 70% as measured with NeuN immunoreactivity in the hippocampus at seven days post-HI, and concomitantly the number of ED1 (CD68) positive cells declined in the ipsilateral hippocampus, thalamus and cortex [148]. Administration of cromolyn, a mast cell stabilizer curtailed significantly brain damage score at one, two and four weeks post-HI (by roughly 70% as measured with Fluoro-Jade B) and a significant reduction in the number of OX-42 (CD11b) positive cells was noted in the ipsilateral thalamus at the same time points [13]. Administration of the COX-2 inhibitor NS398 diminished significantly brain atrophy (assessed via ipsi:contralateral brain weight ratio, degree of neuroprotection ranging between 57 and 79% (two and six weeks post-HI), restored some HI-induced neurobehavioral defects and those effects were associated with a qualitative reduction in single Iba1 and CD68 immunoreactivities in the ipsilateral cerebral cortex at three days post-HI [146]. The exacerbation of neonatal HI-induced brain injury reported after treatment with Fingolimod did not affect significantly Iba1 protein levels one-week post-HI [21]. Cikla et al. [108] carried out the most detailed examination of the microglial phenotype after minocycline treatment using a combination of flow cytometric and immunohistochemical analyses. Mice, subjected to HI at either P9 (mouse model of neonatal HI) or P30 (mouse model of juvenile HI), were injected twice with minocycline at two and 24 h after surgery. In the HI-exposed neonatal mice, the total microglial count (CD11b/CD45 double positive cells) and the percent of activated microglia (CD45^med+^/total) increased in the ipsilateral hippocampus two days post-HI, and then in the ipsilateral cortex and striatum nine days post-HI. The same spatio-temporal rise in microglia was observed in HI-exposed juvenile mice, although not as strong as that observed in HI-exposed neonatal mice. In the mouse model of neonatal HI, minocycline reduced microglial counts (total and percent activated) at both days post-surgery in all regions considered; in the mouse model of juvenile HI, minocycline also impacted microglial counts in the hippocampus two days post-HI, but not at nine days post-HI in cortex and striatum. Surprisingly, long-term neuroprotection was only observed in HI-exposed juvenile mice, but not in HI-exposed neonates. Authors hypothesized that this differential response to treatment was linked to the persistent microglial activation observed in the striatum and cortex of treated HI juvenile mice, thereby suggesting a positive correlation between microglial activation and beneficial outcome. Thus, while the four aforementioned studies suggest a correlation between treatment-associated neuroprotection and a decline in the number of microglia in the ipsilateral hemisphere from treated animals in comparison to that from untreated animals, the latter study actually proposes a link between neuroprotection and persisting microglial activation. The role of microglia in the early phase after neonatal HI was recently addressed in murine models of neonatal HI using genetic and pharmacological based tools to deplete microglia [149, 150]. In [149], microglia depleted male mice (generated by conditional deletion of *Cx3cr1* induced by tamoxifen) subjected to HI at P10 displayed an aggravated neuropathology and astrocyte reaction three days after injury, and overall lower levels of TGF-β and IL-10 in the ipsilateral hemispheres. These data suggest that microglia and specific cytokines play a neuroprotective role in the early phase post-injury. In contrast, in [150], PLX3397 (Plexxikon) was administered orally from P4 to P11, and mouse neonates were subjected to HI at P9. Two days after HI, a significant amelioration in neuropathology was observed, accompanied by a reduction in brain infiltration of circulating immune cells and an improved sensorimotor function, overall suggesting that in the early phase post HI, CSF1R mediated pathways exert a detrimental role in the pathogenesis of the disorder. Reasons underlying such opposing results are unclear but may be linked to differences in HI model (P10 mice, 1 h hypoxia in [149] versus P9 CD1 mice, 30 min hypoxia in [150]) and to the fact that CSF1R inhibition has unspecific effects, as reported in [151]. Another recently reported issue associated with the use of PLX3397 is the actual silencing of typical microglial genes such as *Iba1* and *Cx3cr1*, rather than a depletion of or a reduction in microglial density [152]. Additional microglia targeting agents to be tested include gadolinium chloride (GdCl_3_), a drug that reportedly can deplete selectively pro-inflammatory microglia [153], as well as others potential depletion tools, such as lipid nanocapsules, or polyamidoamine dendrimers as reported in [154].

### Therapeutic Benefit of Stem Cell Transplantation in the Rodent Model of Neonatal HIE: Is Microglia Involved?

**Fig. 2:**
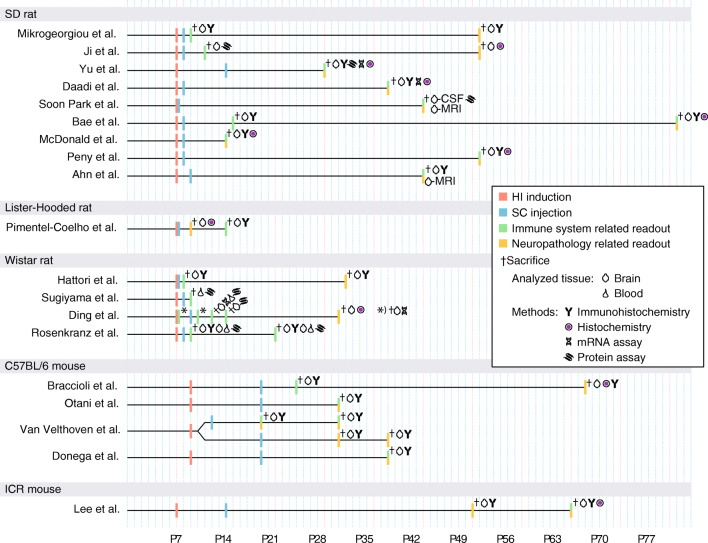
Study designs summarized from Table 4, illustrating large variability in time points of SC transplantation and neuroinflammation related readouts

**Fig. 3:**
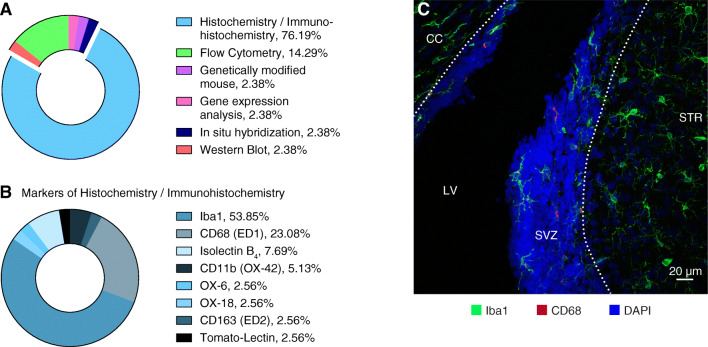
Microglial phenotype-Tools and markers used in the rodent model of neonatal HIE, based on all studies in Tables 1, 2, 3 and 4. Pie charts showing (**A**) the frequency of technical approaches, in percentage of all methodologies and (**B**) the immunohistochemical markers used for the detection of microglia, in percentage of all markers. Of note stainings with Iba1 include single or double stainings (e.g. Iba1 with CD68 or with iNOS). (**C**) maximum image projection from a P30 rat exposed to HI and WJ-MSC treatment as an example of Iba1 and CD68 double immunostaining. CC: corpus callosum, LV: lateral ventricle, SVZ: subventricular zone, STR: striatum, dotted line marks SVZ area

Immune modulation is often put forward as a major mechanism of SC mediated therapeutic benefit. We could find 19 studies—from 12 distinct research laboratories—that reported on SC transplantation in the rodent model of neonatal HI and examined changes in neuroinflammation or in peripheral inflammation post-treatment (see Table 4). A quick glimpse at the table indicates that each study used its own experimental protocol, thus rendering comparison of results between studies difficult. The only common point between these studies is that a single injection of stem cells was used. Variability is otherwise detected at all experimental steps, starting from the rodent model of neonatal HI, through the treatment (type/source, dose and route of SC transplantation), and to the evaluation of neuroinflammation and neuroprotection, as depicted in Figs. 1, 2 and 3. Even when the same treatment protocol is used, variation in other parameters eventually can lead to contrasting outcomes, as illustrated in the two following studies by the same group. In the study of McDonald et al. [155] duration of hypoxia was 180 min and readout endpoint was seven days post-HI, while in the study of Penny [156] these parameters were 90 min and 43 days post-HI. Perhaps mainly due to longer exposure to hypoxia in the first study, deficits in the negative geotaxis test were observed seven days post-HI in the untreated HI-injured neonates, but not in the second study at 43 days post-HI. Stem cell treatment (one million human UCB mononuclear cells transplanted i.p. 24 h after HI) almost fully protected animals from brain damage in the first study, and this was accompanied by an improvement in negative geotaxis test and a significant reduction in the number of Iba1+ cells in the frontal motor cortex. In the second report, stem cell treatment did not limit HI-induced brain damage and an increase in the number of Iba1 activated microglia was observed in the somatosensory cortex, but not in the motor cortex. Thus, experimental variability eventually complicates interpretation.

**Table 4:** Impact of cell therapy on inflammation—preclinical studies

Reference	Species and Model of HI	Cell Therapy	Dose [cells/g BW or BrW]	Cerebral inflammation in the IL hemisphere post-treatment and serum inflammation vs HI-vehicle treated	Degree of neuroprotection	Behavioral outcome HI-SC vs HI-vehicle treated	SC engraftment in the brain parenchyma
Pimentel-Coelho P et al., 2010 PMID: 19296724	Lister-Hooded rat, male 90 min 8% O_2_ Sacrifice time points: 2 and 7 d	3 h route: i.p. 2 × 10^6^ human UCB mononuclear cells labeled with Far Red DDAO-SE.	133′333	Significant reduction in the number of CD68^+^ cells in the cortex 7 d post-HI.	~28% (*) neuroprotection in the striatum 2 d post-HI whereas n.s. changes in the cortex based on Fluoro-Jade C stainings.	Significant improvement in sensorimotor reflexes (cliff aversion reflex, negative geotaxis reflex) by hUBC transplantation 4 d, but not 7 d post-HI.	yes, few human cells detected 2 d post-HI in the IL cortex and striatum, not in CL hemisphere.
Park W et al., 2015 PMID: 25816095	Sprague-Dawley rat, males 120 min 8% O_2_ Sacrifice time points: 35 d	6 h route: i.c. in IL ventricle 1 × 10^5^ human UCB derived MPIO-tagged MSCs with and without hypothermia	100′000	Significant reduction in ED1 (CD68) immunointensity in the penumbra area and reduced CSF levels of IL-1α, IL-1β, IL-6, TNFα observed only in the group of combined hypothermia and MSC transplantation.	29% (*) neuroprotection for MSC only, and 43% (**) neuroprotection for MSC+ hypothermia at 35 d post-HI by MRI.	Significant improvement in sensorimotor function (rotarod test) at P40 and P41 only upon combined treatment of MSCs and hypothermia. Results of cylinder test unclear.	yes, MSCs detected at P42 along the ischemic boundary area. No co-labeling with GFAP or NeuN detected.
Hattori T et al., 2015 PMID: 25720519	Wistar/ST rat 60 min 8% O_2_ Sacrifice time points: 1 and 24 d	6 h route: i.p. 1 × 10^7^ human UCB mononuclear cells	666′667	Significant reduction in the number of ED1^+^ (CD68) cells in the hippocampal dentate gyrus at 1 d post-HI.	No neuroprotection in cortex, corpus callosum and hippocampus at 24 d post-HI based on NeuN stainings.	No significant differences in motor (gait analysis) and learning (active avoidance test) between sham, HI-vehicle and HI-treated animals.	not determined
Mikrogeorgiou A et al. 2017 PMID: 28273662	Sprague-Dawley rat 60 min 8% O_2_ Sacrifice time points: 2 and 43 d	24 h route: i.v. in CL external jugular vein 1 × 10^5^ rat GFP-dedifferentiated fat cells (DFAT)	6′667	Significant reduction in the number of ED1^+^ (CD68) cells in the hippocampus and temporal cortex at 2 d post-HI.	No significant neuroprotection at 43 d post-HI based on MAP2 stainings.	- No significant improvement in sensorimotor tests (rotarod and cylinder test), but trend towards beneficial impact. - No deficits in learning in sham, HI-vehicle and HI-treated animals (novel object recognition test).	not determined
Daadi M et al., 2010 PMID: 20075340	Sprague-Dawley rat 90 min 8% O_2_ Sacrifice time points: 30 d	24 h route: i.c. in IL hemisphere 3 × 10^5^ human NSPCs carrying fLuc and eGFP reporter genes	300′000	Significant increase in the number of Iba1^+^ cells in the striatum, and n.s. trend towards an increase in the cortex. Significant increase in mRNA expression of DCX, CXCR4, FGF2, GDNF, NTN, IGF1, MBP and Olig2.	No significant neuroprotection 4 w post-HI based on cresyl-violet stainings.	Significant improvement in sensorimotor function (cylinder and rotarod) at 1 month post-HI.	yes, hNSCs detected up to 21 d post-HI in the IL hemisphere. Co-labeling with nestin, DCX, TuJ1, GAD and GFAP.
Ji G et al., 2015 PMID: 26255634	Sprague-Dawley rat 120 min 7.8% O_2_ Sacrifice time points: 4 and 43 d after HI	24 h route: intranasal 3 × 10^5^ PKH-26 labeled human NSPCs	300′000	Significant reduction in IL-1β, phosphorylated I*κ*B*α,* cytosolic NF-*κ*B p65 protein levels, and augmentation in nuclear NF-*κ*B p65 at 4 d post-HI.	38% (****) neuroprotection 43 d post-HI based on cresyl-violet stainings.	- Significant improvement in sensorimotor functions (righting reflex, gait, grid walking) early but not late after transplantation. - Significant improvement in sociability (social choice test at 28 and 29 day). - Significant improvement in learning (Morris water maze at 35–40 days).	yes, hNSCs detected 48 h post-HI in the olfactory bulb, cortex, corpus callosum and hippocampus and also 43 d post-HI in the IL hemisphere. Co-labeling with GFAP and NeuN. Some hNSCs also found in CL hemisphere.
Bae S et al., 2012 PMID: 22524897	Sprague-Dawley rat 90 min 8% O_2_ Sacrifice time points: 1 and 10 w post-HI	24 h route: i.v. in external jugular vein 1 × 10^7^ human UCB mononuclear cells +cyclosporine A for 1 month	666′667	Significant increase in the number of Iba1^+^ cells in the periventricular striatum 1 w post-HI, however no difference 10 w post-HI.	No significant neuroprotection 10 w post-transplantation based on H&E stainings.	- No deficits in basic motor function observed in sham, HI-veh or HI-treated groups. - Significant improvement in sensorimotor function (cylinder test) and learning (passive avoidance). Marginal amelioration in depression and anxiety (*p* < 0.06).	yes, human cells detected 1 w post-HI in IL hemisphere around ventricle and co-staining with nestin and DCX. Few cells detected 3 and 10 w post-HI.
Rosenkranz K et al., 2013 PMID: 23123184	Wistar rat 80 min 8% O_2_ Sacrifice time points: 2 and 14 d	24 h route: i.p. 1 × 10^7^ human derived UCB mononuclear cells	133′333	- Significant reduction in CD68 (evaluated by IHC and western blot) in IL hemisphere at 2 d post-HI. - Significant reduction in serum levels of Il-1α, and no impact on Il-1β and TNFα at 2 d post-HI. - No significant differences in serum levels of Il-1α, Il-1β and TNFα between all groups 14 d post-HI.	not quantified	not performed	not determined but previously demonstrated by the same group up to 6 w after transplantation (Geissler et al., 2011)
McDonald C et al., 2018 PMID: 29454374	Sprague-Dawley rat 180 min 8% O_2_ Sacrifice time points: 7 d	24 h route: i.p. either: - 1 × 10^6^ human UCB mononuclear cells, Or UCB cell subtypes: - 2 × 10^5^ endothelial progenitor cells (EPCs), - 2 × 10^5^ T regulatory cells (Tregs), - 2 × 10^5^ monocytes	66′667 (hUBCs) 13′333 (EPCs/Tregs/monocytes)	- Significant reduction in the number of Iba1+ activated microglia in the injured frontal motor cortex after treatment with hUCB mnc, Tregs and EPCs, but not with monocytes. - Significant reduction in brain infiltration of CD4+ T cells by all treatments. - Restoration of CD4+ cell count to sham levels by EPC treatment. - Transplantation of either cell type leads to a CD4+ IFN-γ + Th1/CD4+ IL-4+ Th2 ratio comparable to sham levels, suggesting a reduction of the pro-inflammatory status.	- Almost 100% neuroprotection based on graph representing cresyl violet and acid fuschin stainings for all tested cell subtypes, but images show still damage in UCB derived mnc and monocytes treated animals. - Number of apoptotic cells in frontal cortex significantly reduced in the EPC-treated group only.	- Significant improvement in motor performance (time taken to turn 180° in negative geotaxis test) with hUCB, Tregs and EPCs treatments, but not with monocytes. - Significant improvement in motor performance (time taken to climb 15 cm board in negative geotaxis test) with EPCs treatment only.	not determined
Penny T et al., 2019 PMID: 30967791	Sprague-Dawley rat 90 min 8% O_2_ Sacrifice time points: 43 d	24 h route: i.p. 1 × 10^6^ human UCB mononuclear cells	66′667	Significant increase in the number of Iba1^+^ activated cells in the somatosensory cortex, but not in the motor cortex. The number of resting Iba1^+^ cells remains unchanged in both regions in all groups.	Trend towards an increase in % brain tissue loss based on H&E stainings. In the somatosensory cortex, exacerbation of neuronal damage in the UCB treated group.	- No deficits observed in negative geotaxis, open field test among sham, HI-untreated and -treated animals. - Deficit in cylinder test in HI-untreated animals at P30, although no significant difference with HI-treated animals. No deficits in cylinder test at P50. - Yet, significant amelioration in long-term and overall behavioral burden scores.	not determined
Sugiyama Y et al., 2018 PMID: 30254603	Wistar/ST rat 60 min 8% O_2_ Sacrifice time points: 2 d	24 h route: i.v. in CL external jugular vein 1 × 10^6^ allogeneic rat GFP tagged and DiR labeled bone marrow-derived MSC (BM-MSC) and adipose-derived stem cells (ADSC)	66′667	- Significant reduction in the number of Iba1^+^/iNOS^+^ cells in the penumbra of the cortex and a trend towards a decrease in the hippocampus and basal ganglia after BM-MSC treatment. This is not observed in the ADSC group. - Reduction in serum levels of CCL2, CCL3, CX3CL1, CXCL1, CXCL2, CXCL3, CXCL10, and an increase in IL-2, G-CSF, IL-12p70, IL-17a and TNFα after BM-MSC treatment. ADSC treatment had no such impact.	Not directly quantified. Significant reduction in the number of active caspase-3^+^ cells in the lesioned hippocampus 1 d post treatment in the BM-MSC group, but not in the ADSC group.	not performed	no brain engraftment, however both SCs are detected in lung and liver 4 and 29 d post-HI.
Ahn S et al., 2018 PMID: 29769612	Sprague-Dawley rat, males only 120 min 8% O_2_ Sacrifice time points: 35 d	2 d route: i.c. in the ipsilateral ventricle 1 × 10^5^ human UCB derived MSCs with and without hypothermia	100′000	- Significant reduction in the number of ED1^+^ (CD68) cells in the penumbra area. - Significant decline in the CSF concentration of IL-1α, IL-1β, IL-6 and TNF-α.	No neuroprotection based on MRI 35 d post-HI.	- Significant improvement in performance in rotarod test only observed on the third consecutive day of testing at 35 d post-HI. - No improvement in the negative geotaxis test.	not determined
Ding H et al., 2017 PMID: 26707403	Wistar rat 150 min 8% O_2_ Sacrifice time points: for cytokine levels: 3 h, 6 h, 24 h, 3 d, and 5 d for neuropathology: 21 d	2 d route: i.c. 5 × 10^4^ rat GFP transduced MSCs, placenta derived	50′000	- Significant reduction in mRNA levels of TNF-α, IFN-γ and IL-17 at 3 d post-HI after treatment, and increase in IL-10 IL brain tissue. - Significant decrease in serum protein levels of TNF-α, IFN-γ and IL-17 at 5 d post-HI, and increase in IL-10 levels.	Not quantified. Brain morphology similar between treated and non treated groups 21 d post-surgery based on H&E staining.	Significant improvement in motor activity (evaluated by hanging wire and vertical pole tests) 9 to 21 d post-HI.	2 d post-HI, MSCs found primarily around injection site; 15 d post-HI few MSCs are detected
van Velthoven C et al., 2010 PMID: 19883750	C57BL/6 J mouse 45 min 10% O_2_ Sacrifice time points: 10, 21 and 28 d	3 or 10 d route: i.c. in the IL hemisphere 5 × 10^5^ mouse bone marrow derived GFP-MSCs	1′666’667	Decreased proliferation of microglia (BrdU^+^/Iba1^+^) in the hippocampus (10 and 21 d post-HI) and in the cortex (21 d post-HI) when MSC administered 3 d post-HI. Proliferation of microglia not examined when MSC administered 10 d post-HI.	- 42% (*) neuro-restoration 21 d post-HI, but none 10 d post-HI, based on MAP2 staining when MSCs are administered 3 d post-HI. - 43% (*) neuro-restoration 28 d post-HI, but none 21 d post-HI, based on MAP2 staining when MSCs are administered 10 d post-HI.	- Significant improvement in motor behavior (cylinder rearing test) at 3 and 10 d post-HI when MSCs are administered 3 d post-HI. - Behavioral outcome not reported when MSCs are administered 10 d post-HI.	yes, among which very few are proliferating.
Lee I et al., 2017 PMID: 28081931	ICR mouse, P7 90 min 8% O_2_ Sacrifice time points: 2, 6 and 8 w	7 d route: i.c. in the center of infarcted region 9.6 × 10^5^ NEUROG2 or GFP transduced human NSPCs	3′200’000	Significant increase in Iba1 immunoreactivity in sensorimotor cortex 8 w post-HI for both treatments, other timepoints not examined.	- No neuroprotection 8 w post-HI based on H&E staining. - Less TUNEL^+^ cells in the IL cortex of transplanted animals 6 w after HI.	-- Significant improvement in sensorimotor function at 3, 5 and 7 w and in neuromuscular function at 5 and 7 w post-NEUROG2-NPC transplantation. - GFP-NPC transplantation has a lesser impact on motor functions.	yes, transplanted human NPCs detected in and around the lesion at 3 and 4 w post-transplantation, but not anymore at 5 w. NEUROG2-NPCs differentiate towards neurons, not astrocytes.
Yu Y et al., 2019 PMID: 30177297	Sprague-Dawley rat 150 min 8% O_2_ Sacrifice time points: 21 d	7 d route: i.v. in cervical vein 1 × 10^6^ human UCB-mononuclear cells (MNC) or 1.5 × 10^4^ human UCB-CD34^+^ cells	33′333 (mono-nuclear) 500 (CD34^+^)	Reduction in mRNA levels of TNFα, TNFR1, TNFR2, CD40, Fas at 21 d post-HI. Also reduction in immuno-fluorescence of p65 subunit of NF-κB and GFAP in ischemic areas. Similar results seen after transplantation of MNCs.	27% (*) neuroprotection for CD34+ and 49% (*) neuroprotection for MNC 21 d post-HI based on H&E staining.	Significant improvement in motor function (elevated body swing test) 21 d post-HI for animals treated with human UCB-CD34^+^ cells. UCB-MNCs transplanted animals not tested.	no detection of the transplanted human cells in the brain.
Donega V et al., 2014 PMID: 25396420	C57BL/6 J mouse 45 min 10% O_2_ Sacrifice time points: 28 d	10 d route: intranasal 2 doses: 1 and 2 × 10^6^ human bone marrow derived PKH-26-labeled MSCs	666′667	- Significant reduction in Iba1 immunointensity in cortex and thalamus 28 d post-HI for both doses. - Significant reduction in GFAP immunointensity in cortex and thalamus 28 d after HI only for the 2 × 106 dose.	- 58% (***) neuro-protection with the 2 × 106 dose based on MAP2 staining. - No significant neuroprotection with the 1 × 106 dose.	Significant improvement in sensorimotor function 21 and 28 d post-HI after hMSCs administration (both doses).	yes, strong PKH-26 signal 11 d post-HI around lesion site in the IL hemisphere; no signal in the CL hemisphere.
Otani T et et al., 2019 PMID: 30120407	C57BL/6 J mouse 30 min 8% O_2_ Sacrifice time points: 3 w	10 d route: intranasal 5 × 10^5^ human amniotic fluid stem cells (hAFS) labeled with PKH-26	1′666’667	Significant reduction in number of Iba1^+^ and GFAP^+^ cells near the ipsilateral hippocampus 3 w post-HI.	65% (*) and 76% (*) neuroprotection based on ipsi/contra ratio and MAP2 stainings respectively.	Significant improvement in sensorimotor function 3 w post-HI, but not observed at 10 d post-HI.	yes, transient engraftment up to 22 d post-HI.
Braccioli L et al., 2017 PMID: 27632779	C57BL/6 J mouse 45 min 10% O_2_ Sacrifice time points: 15 d and 56 d	10 d route: i.c. in IL hippocampus 1 × 10^5^ mouse PKH-26 labeled NSPCs	333′333	Significant reduction in Iba1 and GFAP immunoreactivity in the IL hippocampus 15 d post-HI.	50% (**) and 35% (*) neuroprotection based on H&E and MAP2 staining, respectively.	Significant improvement in performance in cylinder rearing test 28 and 56 d post-HI.	yes, transplanted NSCs detected around hippocampus and a fraction differentiated towards neuroblasts 13 d post-HI. No co-labeling with nesting and GFAP.

All sacrifice time-points are given in post-HI. Unless otherwise mentioned, HI induction was performed at P7 and P9 in rats and mice, respectively

^✦^ To facilitate comparison between studies, doses of SCs were converted into number of cells per gram body weight for i.p. and i.v. assuming 15–17-30 g BW for P8–10-14 rat neonates and 5–6-7.5 g BW for P10-P12-P16 mouse neonates, and into number of cells per gram brain weight (gBrW) for i.c. and i.n. assuming a 1 g and a 0.3 g brain weight for a P7 rat and a P9 mouse neonate respectively

**Does stem cell therapy change the microglial phenotype after neonatal HI? If so, how? Is it correlated with neuroprotection and functional outcome?**

Six studies reported a reduction in Iba1-related immunohistochemical parameters—such as number of activated cells, immunointensity and proliferation—in the cortex, the frontal motor cortex, the penumbra of the cortex, the hippocampus, the thalamus at 1-, 5-, 6-, 10-, 14-, 18- and 21 days post-transplantation. This observation was not correlated with a particular cell type, dose, route or time of transplantation (rat/mouse/human bone marrow-BM derived mesenchymal stem cells-MSCs, human amniotic fluid stem cells, mouse neural stem cells, human umbilical cord blood-UCB derived mononuclear cells, T regulatory cells-Tregs, endothelial progenitor cells-EPCs and monocytes, injected i.p., i.v., i.c. or i.n., doses of 13′333 or 66′667 cells per gBW and 333′333–666′667–1′666′667 cells per g brain weight-BrW, injected either 24 h, three or ten days post-HI). Behavioral outcome was evaluated in five studies, and significant improvements in sensorimotor function were reported (assessed in the cylinder rearing test or negative geotaxis test), with some nuances: for instance, amelioration in sensorimotor function was found transient in one report, being detected at three weeks post-HI but not at ten weeks post-HI [157]. Also, the individual cell subtypes of human UCB had differential ability to affect HI-induced deficits in the negative geotaxis test, with UCB derived monocytes being ineffective, in contrast to UCB derived EPCs [155]. The degrees of neuroprotection (assessed with cresyl violet, H&E, MAP2 stainings, ipsi/contra area ratios) were variable, ranging from none to 42%, 58%, 70% to almost full neuroprotection.

Six studies reported a reduction in the CD68 microglia/macrophage activation marker (levels in whole ipsilateral hemisphere, number of positive cells or immunointensity) in the cortex, temporal cortex, hippocampus and dentate gyrus, penumbra area at 1-, 7- and 35 days post-HI. This was again independent of a particular cell type, dose, route or time of transplantation (human UCB derived mononuclear cells and MSCs, or rat dedifferentiated fat cells, injected i.p., i.v. or i.c. either three, six and 24 h post-HI at doses of 6’667, 133′333 or 666′667 cells/gBW and 6′667 cells/gBrW). Assessment of behavior, performed in four studies, revealed significant improvement in sensorimotor function (cliff aversion and negative geotaxis reflex) in only one study, but this effect was transient, being observed four days post-HI, but not seven days post-HI [158]. In two studies, either sensorimotor function was not improved [159], or only trended towards improvement [160]. In two studies, HI-untreated animals did not show any deficits in motor function (gait analysis) [161], or learning (active avoidance test and novel object recognition test), thus rendering the assessment of therapeutic benefit of SC unattainable [160, 161]. Absence of obvious deficits among HI untreated animals versus their sham counterparts was also anecdotally reported in [156], suggesting that some tests may be insensitive or that HI animals could be less affected. Neuroprotection was either detected and significant [158, 159], although in one report restricted to the striatum [158], or absent [160, 161].

Conversely, four studies reported a significant elevation in Iba1-related immunohistochemical measurements (immunoreactivity/overall cell count of cells or of activated cells) in the striatum, periventricular striatum, somatosensory cortex at 7-, 29-, 43- and 49 days post-transplantation. In two independent studies, this elevation was reported to be transient [162], and region-specific [156]. Variability in sources, (human/mouse NSCs or human UCB derived mononuclear cells) routes, i.c. (two studies), i.v. (one study) and i.p. (one study), timing of transplantation, 24 h (three studies) or seven days (one study) post-HI, and doses (66′667 or 666′667 cells/gBW for i.p. and i.v. and 300′000 or 3′200’000 cells/gBrW for i.c. route) excludes a relation between microglial phenotype after SC treatment and any of these factors. Concomitantly, a significant improvement in sensorimotor functions was clearly observed in three studies, the report of Penny [156] being peculiar because HI-untreated animals did not show deficits in individual tests but the calculation of an overall behavioral burden score did reveal an improvement in the HI SC treated group. A common finding between these four studies is that neuroprotection, based on H&E or cresyl violet brain stainings, was not observed. In [156], an exacerbation of neuronal damage was even observed in the sensorimotor cortex after SC treatment. Nevertheless, the number of studies is too low to conclude on a relationship between an increase in Iba1-related measurements and SC associated amelioration in sensorimotor function.

Finally, three studies reported absence of modulation of Iba1-associated measures, such as count [155, 163], or CD68 immunointensity [159] after stem cell transplantation performed at either six or 24 h post-HI. In [163], rat adipose derived stem cells injected i.v. 24 h post-HI did not impact Iba1 microglial count or activation status in the cortex, hippocampus, and basal ganglia or serum chemokine/cytokine levels. The treatment also did not affect the number of apoptotic cells in the hippocampus, and was actually associated with an increased mortality, thus demonstrating a detrimental effect of these particular cells. In [155], i.p. transplantation of human UCB derived mononuclear cells or individual cell subtypes (at 24 h post-HI) show that transplantation of monocytes did not impact the number of activated Iba1 cells in the cortex, and neither neuroprotection nor behavioral improvement were detected. I.c. transplantation of human UCB derived MSCs at 6 h post-HI was not associated with changes in CD68 immunointensity in the HI penumbra area, but neuroprotection was observed and HI-induced deficits in sensorimotor function remained. Nevertheless, combination of this treatment with hypothermia led to a reduction in CD68 immunointensity, together with a better neuroprotection and significant behavioral improvement [159].

Altogether, twelve studies reported a reduction in the activation of microglia upon SC administration, accompanied or not by a concomitant reduction in other cerebral or serum inflammation parameters. In some reports, but not all, this observation was accompanied by some degree of neuroprotection and/or improvement in behavioral outcome. In contrast, four studies reported an increase in these parameters typically accompanied by an improvement in behavioral outcome but not associated with significant neuroprotection. Absence of microglia modulation was also associated with absence of therapeutic benefit. Thus, overall the phenotype of microglia is modulated by SC transplantation, but both—lessened or augmented activation—phenotypes can be associated with a therapeutic benefit. Nonetheless, a major caveat in these studies is that the evaluation of the microglial phenotype relied mostly on single immunohistochemical stainings, which remains insufficient to fully capture the complexity of microglial response after treatment. Future studies could address it more precisely (using a combination of double immunostainings, and/or additional techniques i.e. flow cytometric, genomic and proteomic approaches) taking into account different developmental times and specific regions. The absence of a standardized methodology to assess changes in the microglial phenotype together with the multiplicity of experimental designs prevent for now any definitive conclusion.

#### Transplanted SCs, microglia and neuroprotection/regeneration: mechanisms of the crosstalk

Benefits of SC transplantation are currently mainly attributed to paracrine (over a short distance, i.e. within the damaged brain) and endocrine-like (over a long distance, i.e. from outside the injured brain) signaling, since evidence for cell differentiation and replacement remains scarce. SCs can migrate and home to the damaged brain (Table 4), as they express receptors for chemotactic cues such as stromal cell–derived factor-1 (SDF-1), but homing in the injured site may not be required to elicit benefits. SCs released factors, soluble or present in extracellular vesicles (EVs, e.g. exosomes, microvesicles) are thought to have pleiotropic activities in the host, including immunomodulatory, antiapoptotic, angiogenic, and neurotrophic effects. A particular interest in the role of EVs shed by exogenous SCs on endogenous microglia has recently emerged, as microglia appear crucial “EV recipient cells” in comparison to other cell types in the brain [164–166]. Until now three studies have investigated the therapeutic potential of bone marrow MSCs derived EVs (administration routes were either i.p., intranasal or intracardiac) in the murine model of neonatal HI, and all reported a reduction in microglial activation, with two studies also reporting functional benefits [167–169], similar to findings of studies using actual MSCs (Table 4). Thus, overall, a complex crosstalk between exogenous SCs, endogenous microglia and neuronal cells mediated by EVs may explain in part their therapeutic potential. A question remains though whether the EVs released by SCs after transplantation are of the same nature of those collected from in vitro cultured and expanded cells, as the diseased environment of the host may impact SCs secretion profile.

## Is Inflammation in HIE and CP Modulated by Stem Cell Treatment? Knowledge from Clinical Studies

The tools allowing to evaluate the immune-system associated changes in living humans after cell-therapy are mainly the measurement of cytokine levels in plasma/serum or cerebrospinal fluid (CSF). Modern imaging techniques listed hereafter may also be useful, although with limitations. Positron emission tomography-computed tomography with the radiotracer ^18^F-fluorodeoxyglucose (^18^FDG PET-CT), which measures glucose metabolism, is a valuable tool, as inflammatory cells are highly glycolytic and foci of increased uptake of ^18^FDG in the brain may be interpreted as sites of inflammation. Brain magnetic resonance imaging with diffusion tensor imaging sequences (MRI-DTI) provide relevant information on brain connectivity, indicative of white matter pathologies. Single-photon emission computed tomography (SPECT) can answer questions on inflammation, and on cerebral blood flow and blood brain barrier injury. These three imaging techniques are nevertheless not specific for immune-related changes, and therefore caution in data interpretation is warranted. In the past decade, clinical trials have investigated mainly safety, feasibility, and efficacy of cell-based therapies in HIE and CP (Table 5). Up to now, among 25 trials, we found three studies reporting on cytokine levels after SC transplantation, two in plasma [170, 171] of CP patients, and one in the CSF of intracerebral hemorrhage (IVH) patients [172]. ^18^FDG PET-CT imaging was also performed in [170, 173] and three additional studies [174–176]; brain MRI-DTI data were reported in [173, 174, 177, 178] and SPECT data in [177]. To the best of our knowledge, studies using PET radioligands targeting the 18 kDa translocator protein (TSPO), a surrogate marker of neuroinflammation (e.g. activated microglia and astrocytes), have not been performed in infants diagnosed with CP or an early HI brain injury. Promising studies in rodent models of adult stroke and in human stroke patients have been reported [179–181]; nevertheless, questions on cellular specificity persist, and insufficient binding affinity of these radioligands is documented in patients harboring polymorphisms in TSPO [182].

**Table 5:** Impact of cell therapy on inflammation—clinical studies

Reference(s)	Trial ID / Country, Phase /Allocation	Start / Last Update	Condition, Participants (Age)	Cell Therapy	Outcome	Investigation of Immune system reported or planned?	Administration of immuno-suppressant(s)
Ahn S. et al., 2018 PMID: 30133179	NCT02274428 Korea, 1 open label	October 2014 / April 2017	IVH (grade 3–4), 9 (23–34 weeks)	Allogenic UCB-MSC low-dose: 5 × 10^6^ cells/kg or high-dose: 1 × 10^7^ cells/kg route: intraventricular	- Intraventricular administration route appears safe in the small study population. - Levels of CSF-IL-6 significantly decline after MSC transplantation. - Baseline CSF-IL-6 is higher in infants who underwent shunt surgery than in infants without shunt placement. - Correlation between baseline levels of CSF-IL-6 and TNF-α and baseline ventricular index.	Reported: CSF cytokines	no
Kang M et al., 2015 PMID: 25977995	NCT01528436 Korea, 2 randomized; double-blind; placebo-controlled	February 2012/July 2012	CP, 34 (6 month - 20 years)	Allogenic UCB-TNC ≥ 2 × 10^7^ cells/kg route: intravenous or intraarterial	- Greater improvement in muscle strength and gross motor performance in the UCB group at 3 and 6 months, respectively. - 18FDG PET-CT shows decreased periventricular inflammation in the UCB group. - Enhanced gross motor function correlates to increased plasma PTX3 and IL-8 levels up to 12 days after treatment in the UCB group. - Increased serum TLR4 1 day after treatment and correlation with muscle strength 3 months post treatment.	Reported: plasma cytokines, serum levels of TLR4, ^18^FDG PET-CT	yes, cyclosporine and solumedrol (=methyl-prednisolone)
N/A	NCT03130816 Korea, 1; 2 open label	April 2017/November 2020	CP, 90 (10 month - 20 years)	Allogenic UCB-TNC ≥2 × 10^7^ cells/kg route: intravenous	N/A	Planned, but not clearly defined	yes
Min K et al., 2013 PMID: 23281216	NCT01193660 Korea, N/A double-blind; randomized controlled	September 2010/March 2012	CP, 96 (10 month - 10 years)	Allogenic UCB-TNC and erythropoietin combination > 3 × 10^7^ cells/ kg 250–500 IU(kg) erythropoietin route: intravenous	- At 3 and 6 months younger children of the UCB + EPO group showed greater improvements than EPO alone and the placebo group for GMPM and BSID-II, in the 3 to 6 months interval additionally also the EPO alone group showed better outcome than the control. - Older children showed significant improvement in the BSID-II score during 0 to 3 months period. - MRI-DTI reveals significant correlation between changes of the FA values and GMPM increment.	Reported: ^18^FDG PET-CT, MRI-DTI	yes, cyclosporine
Huang L et al., 2018 PMID: 29637820	Ethical approval No. 2010–06 China, N/A randomized, placebo-controlled	September 2010/September 2015	CP, 56 (3–12 years)	Allogenic UCB-MSC 5 × 10^7^ route: 4 x intravenous	- Significant improvement in GMFM-88 and CFA noted at 3, 6, 12 and 24 months when compared to the control group. - Immunologic tests performed to monitor rejection before and after cell administration (IgA, IgM, C3, C4, RF, CRP, anti-streptolysin) within normal ranges. - No cerebral structure improvement observed in routine MRI.	Reported: serum immune markers of rejection	no
Gu J et al., 2020 PMID: 32014055	ChiCTR-TRC-1800016554 China, N/A randomized, placebo-controlled	June 2018/July 2019	CP, 39 (2–12 years)	Allogenic UC-Wharton’s Jelly derived MSC 4.5–5.5 × 10^7^ route: 4 × intravenous	- Significant improvements of GMFM, CFA and ADL at 1, 3, 6 and 12 months after treatment. - Regional increase in standard uptake value of glucose measured in 3 out of 5 patients of the treatment group.	Reported: ^18^FDG PET-CT	no
Sharma A et al., 2015 PMID: 25788947	NCT01978821 India, N/A open label	August 2010/October 2018	CP, 40 (17 month - 22 years)	Autologous BM-MNC 1 × 10^7^ route: intrathecal	- Subgroup analysis shows improvement for diplegic and quadriplegic CP patients superior to that in the miscellaneous group of CP patients. - FDG PET-CT scans in 6 patients show metabolic changes correlating to clinical improvements.	Reported: ^18^FDG PET-CT	yes, methyl-prednisolone
Rah W et al., 2017 PMID:28109298 Park K et al., 2017 PMID: 28289643 Koh H et al., 2018 PMID:29780293	NCT02983708 Korea, 1; 2 randomized; double-blind cross-over	August 2011/September 2014	CP, 57 (2–10 years)	10 μg/kg G-CSF for 5 days + Autologous mPB-MNC either 1 or 7 month after cryopreservation > 1 × 10^8^ cells/kg route: intravenous	- Neurodevelopmental improvement after G-CSF infusion, irrespective of the cell infusion or placebo. - The group receiving cell therapy seven months after G-CSF had higher motor-functional improvement, whereas the group receiving cells one month after G-CSF had a significant increase of FA values in MRI. - No cytokine differences observed between the intervention arms. - However, in clinical responders, plasma levels of IL-6 and G-CSF were significantly higher than in non responders one month after G-CSF treatment, and levels of BDNF and IGF-1 were significantly lower.	Reported: plasma cytokines, MRI-DTI, ^18^FDG PET-CT	no
Lee Y et al., 2012 PMID: 22443810	N/A Korea, N/A open label	N/A	CP, 20 (2–20 years)	Autologous UCB-TNC 5.5 × 10^7^ cells/kg route: intravenous	- Safe and feasible. - Functional benefit in some CP patients that could be linked to MRI-DTI and SPECT evaluations.	Reported: MRI-DTI; SPECT	no
N/A	NCT02866331 Korea, 2 randomized; double-blind;	July 2016/December 2018	CP, 88 (2–10 years)	Autologous UCB-MNC and G-CSF (10 μg/kg/day) (Leucostim) route: intravenous	- No data published - Based upon feasibility and safety (https://www.ncbi.nlm.nih.gov/pubmed/22443810)	Planned: MRI-DTI, peripheral blood CD34+ cell counts, neurotrophic factors/anti-inflammatory cytokines.	no
Sun J et al., 2017 PMID: 29080265	NCT01147653 USA, 2 randomized, place-controlled	June 2010/November 2012	CP, 63 (1–6 years)	Autologous UCB-TNC 1–5 × 10^7^ cells/kg Categories of low and high infused doses Low: <2 × 10^7^/kg, *n* = 16 High: ≥2 × 10^7^/kg, n = 16 route: intravenous	- No significant difference in change in GMFM-66 score between intervention and control group 1 year after infusion in the whole study cohort. - Infants who received higher cell doses (≥2 × 107/kg infused) demonstrate superior gains in both whole brain connectivity and motor function 1 year after infusion of cord blood cells.	Reported: MRI-DTI Planned: Correlation between clinical response and RNA expression of inflammatory cytokines in transplanted cord blood cells	yes, methyl-prednisolone
Liu X et al., 2017 PMID: 28235424	ChiCTR-TRC-12002568 China, 1; 2 randomized, rehabilitation-controlled	October 2012/October 2015	CP, 105 (4–10 years)	Autologous BM-MSC Autologous BM-MNC 1 × 10^6^ cells/kg Control group: Bobath therapy only route: 4 × intrathecal	- BM-MSC administration improves significantly the total scores of GMFM and FMFM after 6 and 12 months when compared to the control group. - BM-MNC administration does not improve these scores in comparison to controls. - BM-MSC transplantation is feasible and safe.	Planned: T lymphocyte subsets determination	no
Luan Z et al., 2012 PMID: 22507684	N/A China, N/A randomized controlled	May 2005/May 2006	CP, 94 (<5–40 month)	NSPC from human aborted fetal forebrain tissue 8–10 × 10^6^ route: intraventricular	- Significantly better GMFM and PDMS-FM in treated group after one year. - Rate of improvement decreased gradually within 3 months of transplantation. - No delayed complications observed.	no	no
review article^✢^	KCT0001429 (CRIS) Korea, 1; 2 open label	August 2006/August 2013	HI, 41 (≥ 36 weeks)	NSPC from human aborted fetal forebrain tissue 25 × 10^7^ route: intracerebral (in the brain injury)	N/A	no	not specified
Cotten C et al., 2014 PMID: 24388332	NCT00593242 USA, N/A open label	January 2008/March 2017	HIE, 23 (12–72 h)	Autologous UCB-TNC 1–5 × 10^7^ cells/dose route: 4 × intravenous	- UCB transfusion is safe and feasible. - Phase II studies are warranted.	no	yes, hydro-cortisone
Gabr et al., 2015 not available	N/A Egypt, N/A randomized, rehabilitation-controlled	June 2008/June 2009	CP, 94 (1–7 years)	Autologous BM-MSC 2 × 10^6^ route: intrathecal	- The MSC injections show short-term safety. - PEDI and Gross motor-function score show no significant improvement.	no	not specified
Mancías-Guerra C et al., 2014 PMID: 24642016	NCT01019733 Mexico, 1 open label	July 2009/January 2011	CP with HI etiology, 18 (1–8 years)	G-CSF (10 μg per kg/day) for 4 consecutive days+ Autologous BM-TNC 13 × 10^8^ route: intravenous and intrathecal	- G-CSF-mobilized autologous BM-TNCs intrathecal and intravenous administration in children with CP is safe. - Possible increase in neurological function.	no	yes, hydrocortisone
Chen G et al., 2013 PMID: 23351389	ChiCTR-TRC-12002056 China, N/A open label, non-randomized, controlled	July 2010/August 2015	CP, 60 (1–21 years)	NSPC-like cells derived from autologous BM-MSC 1–2 × 10^7^ route: intrathecal	- NSC-like cells are safe. - GMFM scores in the transplantation group significantly higher 3 and 6 months post-treatment compared with the baseline scores. - No significant increase in GMFM in the control group. - No significant increases in the language quotients at months 1, 3, and 6 post-treatment when compared with the baseline quotients in both groups.	no	not specified
Romanov Y et al., 2015 PMID: 25791070	Roszdravnadzor, Permission #2009/387 Russia, N/A open label	January 2011/December 2013	CP, 80 (1–12 years)	Allogenic UCB-TNC 2.5 × 10^8^ route: 1–6 × intravenous	- In 3–36 months follow-up, no adverse effects observed. - More UCB-infusions correlate positively with neurological status and cognitive functions.	no	prophylactic anti-allergic drug, Clemastine
N/A	NCT01639404 Korea, N/A open label	July 2012/August 2013	CP, 17 (6 month - 20 years)	Allogenic UCB-TNC route: intravenous or intraarterial	N/A	no	yes, non-myeloablative immuno-suppressant
N/A	NCT01988584 USA, 2 randomized, placebo-controlled	November 2013/May 2020	CP, 20 (2–10 years)	Autologous BM-MNC or autologous UCB-TNC route: intravenous	N/A	no	not specified
Tsuji M et al., 2020 PMID: 32165664	NCT02256618 Japan, N/A open label	August 2014/October 2019	HIE, 6 (12–72 h)	Autologous UCB-TNC 1.4–10.9 × 10^8^ cells in total route: 3 × intravenous	UCB transfusion is safe and feasible.	no	yes, hydro-cortisone
	NCT03078621 Jordan, 1; 2 open label	September 2016/March 2020	CP, 50 (2–12 years)	Autologous BM-derived stem cells and MSC route: intravenous and intrathecal	N/A	no	not specified
Wang X et al., 2013 PMID: 24100132	ChiCTR-TRC-10000928 China, N/A open label	N/A	CP, 52 (6 month - 15 years)	Autologous BM-MSC 2 × 10^7^ route (2 protocols): - Patients ≥5 years or with head circumference ≥ 50 cm: 2 × intrathecal +1 × intraparenchymal - Patients <5 years or with head circumference < 50: 4 × intrathecal	- Procedure is safe and feasible. - The GMFM-66 percentile used as a control index indicates a significant improvement at 1, 6 and 18 months after transplantation. - The intraparenchymal group did not profit more from transplantation.	no	not specified

✢ http://www.neo-med.org/journal/view.php?number=777, publication in Korean language

Abbreviations

N/A = not available

For pathologies

CP = cerebral palsy; HIE = hypoxic ischemic encephalopathy; IVH = intraventricular hemorrhage

For cells

UC = umbilical cord; UCB = umbilical cord blood; BM = bone marrow; TNC = total nucleated cells; MNC = mononuclear cells; MSC = mesenchymal stem cells; NSC = neural stem cells; NPC = neural progenitor cells; mPB-MNC = mobilized peripheral blood mononuclear cells

Notes

• all injected cells are of human origin

• Cell counts for administration are displayed per administration

For functional assessments.

^18^FDG PET-CT = 2-deoxy-2-[fluorine-18]fluoro- D-glucose integrated with computed tomography; MRI-DTI = magnetic resonance imaging (MRI)-diffusion tensor imaging; SPECT = single-photon emission computed tomography; FA = fractional anisotropy.

CFA = comprehensive functional assessment; PEDI = Pediatric evaluating self care, mobility and social function; GMPM = Gross Motor Performance Measure; FMFM = fine motor function measurement; BSID-II = Bayley Scale of Infant Development-II; PDMS-FM = Peabody Developmental Motor scale-Fine Motor; ADL = activities of daily living.

For other measures.

CSF = cerebrospinal fluid; IL = interleukin; PTX3 = pentraxin 3; TLR4 = Toll-like receptor 4; G-CSF = granulocyte colony-stimulating factor.

### General Considerations on SCs Used in Clinical Trials

**Fig. 4:**
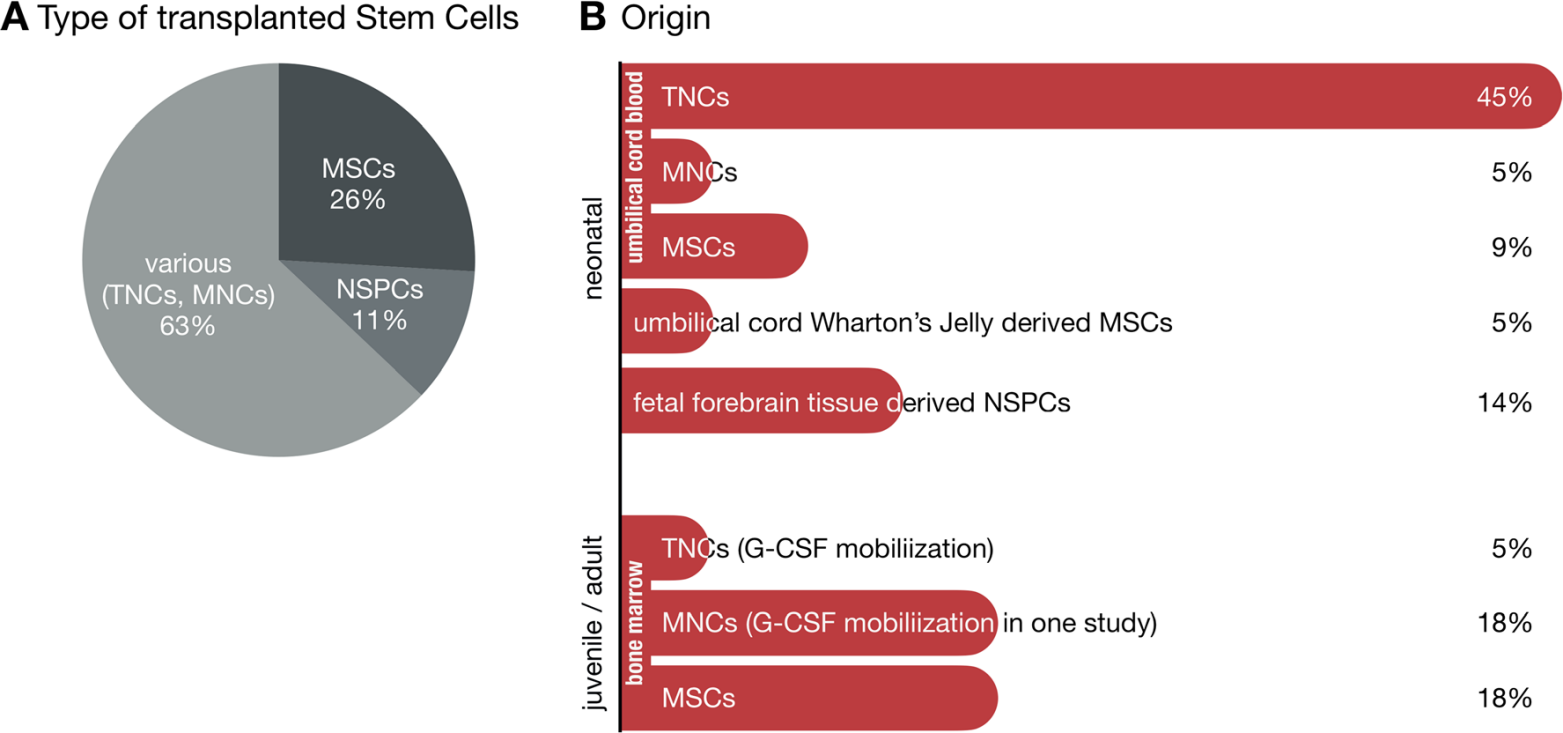
Umbilical cord blood derived cells are most frequently used in human clinical trials. (**A**) Pie chart showing percentage of the types of stem cells tested in trials. (**B**) Bar graph displaying origin of the transplanted stem cells and corresponding percentages. For both charts, percentages were calculated from all studies listed in Table 5

In clinical trials, umbilical cord blood (UCB) derived cells (i.e. total nucleated cells-TNCs, mononuclear cells-MNCs and MSCs) are the most frequently used cells (Fig. 4). SCs from neonatal tissue may display improved features in comparison to those isolated from adults, for instance MSCs derived from neonatal tissue display improved capacity for proliferation, expansion and engraftment in comparison to adult MSCs [183]. Cells from birth related tissue (UC, placenta) also raise no ethical issues; practically, they can be easily harvested and banked and are therefore rapidly available for both autologous or allogenic use in human patients. Alternative strategies for endogenous brain repair also include the mobilization of endogenous BM derived SCs to the bloodstream; this is typically achieved with granulocyte colony stimulating factor (G-CSF) but additional methods are being tested, for instance co-injection of NOx-12, a compound that acts in a similar fashion to G-CSF, with SDF-1 to promote recruitment of the cells and favor paracrine effects at site of injury, as reported in a model of retinal degeneration [184].

### Immune related findings in clinical trials

Allogenic infusion of UCB-TNCs in CP patients resulted in elevated levels of plasma pentraxin 3 (PTX3), interleukin-8 (IL-8) and in an increased number of blood cells expressing Toll-like receptor 4 (TLR4) during a consecutive short period after infusion. The elevation in PTX3, IL-8 and TLR4 levels correlated with a better Gross Motor Function Measure (GMPM) outcome up to six months after treatment [170]. The role of the cytokine PTX3 is not fully understood but several studies suggest an anti-inflammatory, protective effect in various conditions [185–187]. IL-8 is a chemokine that promotes chemotaxis towards the injured area and was shown to have an angiogenic effect [188]. A study further investigating the role of PTX and TLR4 after allogenic UCB infusion in CP is currently pending (NCT03130816). In that same study, two weeks after treatment, a decreased activity in ^18^FDG PET-CT in the white matter of the occipital and temporal areas was detected. The authors interpreted this finding as an anti-inflammatory effect of UCB-TNC, as these areas are typically inflamed in PVL, a significant cause of CP. Increased activity was also observed in cortical areas, which the authors interpreted as a possible correlate of improved motor function. In a previous study, the same group had investigated the effect of allogenic UCB infusion in combination with EPO [174]. They found significant fractional anisotropy (FA) value increments in all measured loci and especially a correlation of the changes in the posterior limb of the internal capsule (PLIC) with the GMPM in the first six months [174]. An additional effect of repeated administration of allogenic UCB-TNC was found in a large open label study including 80 patients [189]. Also, Sun et al. found a dosage dependent effect after autologous UCB reinfusion with an improved brain connectivity analyzed via MRI-DTI, supporting the notion that paracrine signaling underlies the UCB effect leading to GMFM scores above those predicted by age and severity in CP patients [178]. Main limitation of the aforementioned studies with allogeneic and autologous UCB-TNC transplantations is the administration of the immunosuppressants cyclosporin and/or methylprednisolone which could explain findings as no cyclosporin/methylprednisolone-only controls are included. Regarding HIE, autologous UCB infusion without prior cryopreservation was safe in a Phase 1 study [190], and feasibility was also demonstrated in cases where cord blood collections were insufficient for banking criteria [191].

The impact of administration of autologous G-CSF mobilized peripheral blood MNCs (mPB-MNCs) on plasma cytokines was also reported in a randomized double-blind crossover study including 16 CP patients [171]. Their neuroregenerative effect was rather marginal, and motor-functional improvements did not differ after placebo or mPB-MNC reinfusion. Interestingly after G-CSF treatment the improvements were significantly higher than during the period of reinfusion of cells. Overall, no significant difference in plasma levels of the following factors, i.e. G-CSF, brain-derived neurotrophic factor (BDNF), vascular endothelial growth factor (VEGF), insulin-like growth factor IGF-1, IL-6, IL-8, IL-10 was observed between the intervention arms. However, in clinical responders, plasma levels of IL-6 and G-CSF were higher one month after G-CSF infusion, and levels of IGF-1 and BDNF were lower, suggesting that these cytokines may be used as prognostic factors in G-CSF trials and that they may be associated with the G-CSF driven neurological improvements. MRI-DTI showed significantly higher FA-values in patients who received cell therapy one month after G-CSF treatment in comparison to those who received it seven months later, suggesting a possible synergetic effect if mPB-MNC are infused shortly after G-CSF, although ^18^FDG-PET did not show the expected differences [171, 173, 192]. In another study enrolling 18 CP patients, mPB-MNC were considered safe for combined intrathecal/intravenous administration with possible benefit on neurological function at six months after infusion. In that study the collection and reinfusion of cells were performed on the same day, four days after G-CSF mobilization [193]. Since G-CSF is an immune system stimulating factor, its contribution to positive effects cannot be ruled out; a clinical trial including a study arm with G-CSF alone is registered (NCT02866331, last updated 2016).

In a clinical study assessing the effect of intraventricular administration of allogenic UBC derived MSC in nine preterm infants with severe intracerebral hemorrhage (IVH), the CSF levels of IL-1β, IL-6, TNF-α, VEGF, TGF-β1/2, BDNF and fibroblast growth factor (FGF) were investigated before and after MSC transplantation [172]. Results showed that the concentration of IL-6 significantly declined after transplantation, while that of other cytokines or growth factors also tended to decline, but not significantly. Of note, the baseline levels of IL-6 were significantly higher in infants with shunt surgery than in infants without, which may confound the results observed after MSC transplantation. A correlation between baseline CSF levels of IL-6 and TNF-α and ventricular index was observed. Authors suggest that these cytokines may be used as markers of early neuronal injury, but also acknowledge the small sample size and the need for further studies. In a trial using autologous BM derived MSC in 40 CP patients, ^18^FDG PET-CT changes were observed correlating to clinical improvement after intrathecal administration [175]. A safety study comparing an intraparenchymal with intrathecal administration of autologous BM-MSCs did not find a superior effect of intraparenchymal versus intrathecal administration, supporting again the hypothesis of paracrine signaling for SC-mediated positive outcome. Of note, the invasive intraparenchymal access was also considered safe [194]. In contrast, one study also testing autologous BM derived MSC in 94 CP patients showed no functional short-term improvements [195]. Allogenic MSC derived from UCB or UC Wharton’s Jelly were also tested in two Chinese studies and showed motor-functional improvements during twelve [176] and 24 months [196] of follow up, respectively. Improvements in cerebral structure could not be observed in MRI-DTI, but ^18^FDG-PET revealed increased glucose uptake in some patients of the treatment group which could support functional improvements, since lowered glucose metabolism can be found in CP patients [197].

Overall, the efficacy of stem cells via the modulation of the immune system at this stage remains hypothetical given the limited number of studies. Immunosuppressive or stimulating growth factors like cyclosporine and G-CSF may play a role in the inflammatory status of the brain or other organs which eventually could confound the results.

## Conclusion

Stem cell-based therapies hold promise as alternative or complementary neuroprotective/regenerative therapeutic strategies for infants diagnosed with neonatal HIE or CP. In a context where these therapies are being advertised by uncontrolled institutes, and understandably, families are eager to offer treatment to their children to ameliorate the persisting neurological disabilities, it is crucial to improve our basic understanding of their mechanism of action to eventually optimize and advance the clinical development of SC protocols.

Our review of the preclinical literature indicates that stem cell treatments can modulate the microglial phenotype after neonatal HI, but a causal link between this immune-related modulation and neuroprotection is difficult to establish, due partly to a limited number of studies, caveats in methodology and highly heterogenous experimental designs. The concept that microglia may be a therapeutic target is appealing [152], but in its current state, requires further research. Clinical data on immunomodulation after SC transplantation in HIE and CP patients remain anecdotical and hold considerable uncertainties that also call for further investigations. Collaborative efforts between researchers and medical practitioners from different fields (neurodevelopment, neuroimmunology, neurobehavior, pediatry, neurosurgery) as well as general guidelines as to how to characterize microglia -similar to those established for macrophages- and various immune related parameters, would advance our understanding of the link between the cerebral immune system, microglia and neuroprotection.

## Data Availability

Not applicable.
